# A study on the "Porter Hypothesis" effect of the regulatory measures of the environmental protection tax law in the post-pandemic era

**DOI:** 10.1371/journal.pone.0304636

**Published:** 2024-05-31

**Authors:** Wei Tao, Jian-ya Zhou

**Affiliations:** 1 School of Finance and Business, Zhenjiang College, Zhenjiang, China; 2 School of Management, Jiangsu University, Zhenjiang, China; Ovidius University of Constanta: Universitatea Ovidius din Constanta, ROMANIA

## Abstract

The implementation of the Environmental Protection Tax Law was a significant milestone in China’s environmental tax reform. The implementation of this law was influenced throughout the three-year period of epidemic prevention and control (from early 2020 to the end of 2022). Heavily polluting enterprises are the primary focus of regulations under the Environmental Protection Tax Law. This study conducts an empirical analysis using a structural equation model, leveraging sample data obtained from heavily polluting enterprises in China. The findings indicate that during the three-year period of epidemic prevention and control, the Porter Hypothesis effect was realized in terms of tax fairness but not in terms of tax rationality. Therefore, environmental tax law reforms should be pursued and tax authorities in China should make vigorous efforts to enhance the rationality of environmental taxation. This would improve the comprehensiveness of the “Porter Hypothesis” effect, fully harnessing the dual functions of environmental protection and the economic driving force embodied by the Environmental Protection Tax Law.

## 1 Introduction

During the three-year period of epidemic prevention and control, China experienced phenomenal evolution in its social and economic sectors. From the onset of the epidemic in Wuhan in early 2020 to the complete recalibration of control policies by the end of 2022, China has navigated through an unparalleled era, akin to a once-in-a-century event. Guided by the Party and the government, Chinese society engaged in a formidable struggle against the epidemic, ultimately mitigating its losses. However, throughout the three years of epidemic prevention and control, the economic and social development faced substantial repercussions and numerous transformations. The outbreak of the new coronavirus crisis has made many enterprises realize the developmental limitations of traditional industries and has hindered the effectiveness of government policies [1]. The effects of such sudden crises are complex, difficult to quantify, and require prompt changes in production patterns and environmental interventions [2]. Despite significant adjustments in epidemic prevention policies, China still grapples with a severe epidemic situation. Hence, it is imperative to delineate and comprehend the economic circumstances and conditions during the three-year period of prevention and control, furnishing a roadmap for future development.

The Environmental Protection Tax Law of China has been in force since January 1, 2018, with a cumulative duration of five years, coinciding with three years of stringent epidemic prevention and control. During these three years, the strategies, approaches, and effectiveness of the implementation of the “Environmental Protection Tax Law” were inevitably subjected to changes. During early 2023, following the adjustments to the epidemic prevention policies, corresponding adjustments should have been made to the implementation plan of the Environmental Protection Tax Law. The implementation of the Environmental Protection Tax Law symbolizes the formal establishment of China’s environmental protection tax system. However, because tax objects are pollutants that are difficult to accurately measure, they pose significant challenges in monitoring. Close cooperation among tax authorities, environmental protection agencies, and other relevant departments is necessary to establish a comprehensive set of tax administration measures to effectively implement the Environmental Protection Tax Law. Additionally, the Environmental Protection Tax Law possesses a certain uniqueness and complexity in its collection and management processes, requiring continuous improvements.

The theoretical foundation of the Environmental Protection Tax Law can be traced back to nineteenth-century economist Arthur Pigou’s theory of Pigouvian taxation. This theory suggests that a country or region can impose taxes on polluters based on the level of harm caused by their pollution, thereby bridging the gap between the private and social costs incurred by polluters [3]. In the 1960s, developed countries began enacting legislation on environmental protection taxes and its collection, establishing relatively mature environmental economic policy systems [4]. However, economic development and environmental protection are not inherently antagonistic. Environmental policy adjustments foster sustainable development, which is indispensable to the advancement of human society [5].

The Environmental Protection Tax Law is an important type of market-based environmental regulation and therefore exhibits the characteristics of the “Porter Hypothesis” effect. Porter (1991) proposed that when businesses or industries are subjected to environmental regulations, it not only enhances environmental protection and social benefits but also improves the profitability of regulated businesses or industries. This is the fundamental concept underlying Porter’s hypothesis [6]. Jaffe (1997) categorized the “Porter Hypothesis” into two types: the “Weak Porter Hypothesis” and the “Strong Porter Hypothesis,” representing two stages of the hypothesis’s development. The “Weak Porter Hypothesis” suggests that environmental regulations can stimulate technological innovations in businesses, but the cost incurred from such innovations does not fully offset the expenses associated with environmental regulations, resulting in no significant growth in a firm’s competitive advantage. The “Strong Porter Hypothesis” argues that environmental regulations not only promote technological innovations in businesses but also enable the innovations to compensate for the cost incurred from environmental regulations, thereby enhancing a firm’s competitive advantage [7].

Heavily polluting enterprises constitute a distinct category of companies characterized by significant environmental pollution arising from the production and application of their products. Consequently, they are the primary targets of regulation under the Environmental Protection Tax Law, making them pivotal subjects of study within the realm of environmental regulation research. Environmental regulations can promote green technology innovations, thereby influencing the energy and environmental performance of heavily polluting enterprises, corroborating Porter’s hypothesis [8].

Some studies suggest that in the first two years of implementing the Environmental Protection Tax Law, a discernible “Porter Hypothesis” effect was observed within heavily polluting enterprises. Cui and Lu (2021) conducted an empirical investigation using a difference-in-differences model and a comprehensive dataset of Chinese A-share listed companies operating as heavily polluting enterprises from 2014 to 2019. Their study robustly tested the impact of the Environmental Protection Tax Law and unveiled compelling findings, whereby the law not only stimulated green investments within heavily polluting enterprises but also exhibited a substantial positive influence on their levels of autonomous technological innovation [9].

However, most studies suggest that there are no discernible “Porter Hypothesis” effect on heavily polluting enterprises in the first two years of implementing the Environmental Protection Tax Law. Chen and Wu (2021), using a difference-in-differences model and sample data from Chinese A-share listed companies from 2011 to 2019, found that ever since the introduction of the environmental protection tax in China, heavily polluting enterprises have been motivated to increase their environmental investments, but it has neither reduced their pollutant emissions nor alleviated the burden of environmental protection taxes [10]. Ma and Guan (2022), based on sample data from Chinese A-share heavily polluting enterprises from 2012 to 2019, found that the collection of environmental protection taxes exhibits a significant “compliance cost” effect but has not demonstrated a significant “innovation compensation” effect, thus indicating an insignificant “Porter Hypothesis” effect [11]. Cao and Tang (2022), utilizing a difference-in-differences model (DID) with sample data from Shanghai and Shenzhen A-share listed companies in the heavily polluting industry from 2015 to 2020, examined the impact of the Environmental Protection Tax Law on the total factor productivity of heavily polluting enterprises. They discovered that the implementation of environmental protection taxes significantly improved the total factor productivity of heavily polluting enterprises. However, this improvement did not result from the innovation compensation effect or the “Porter Hypothesis” effect of environmental protection taxes. Instead, it has been attributed to the enhancement of resource allocation efficiency in heavily polluting enterprises [12].

From a global perspective, there is a consensus on steering enterprises towards environmentally friendly practices through environmental regulations to enhance ecological conditions. The scarcity of resources and environmental crises require economic development to follow sustainability and secure circular economy with the help of green transformation policies [13]. European countries also actively promote the construction of environmental protection tax laws and study environmental regulations and enterprise performance during the implementation process. Souguir et al. (2024) believe that though implementation of tax and environmental protection incentives can promote the environmental performance of enterprises, but it may simultaneously induce tax evasion by enterprises [14]. In a study of G7 carbon emissions, Khaddage-Soboh et al. (2023) found that environmental regulations and taxes are conducive to reducing carbon emissions and optimizing environmental performance; however, they questioned the role that environmental taxes can play in CO_2_ emissions [15]. In a study in the UK, Zhou et al. (2023) found that environmental taxes have a significant positive effect on promoting sustainable economic development, provided that they effectively regulate the green behavior of enterprises [16]. Onwe et al. (2023) studied the effect of environmental taxes on ecological sustainability in G7 countries and found that the implementation of environmental taxes could encourage enterprises to change their energy utilization structure and strengthen the research and development of green technologies owing to their profit-driven characteristics [17]. Germany, France, Italy, and the United Kingdom are taking measures to address environmental sustainability using environmental taxes to mitigate the negative environmental externalities of enterprises, thereby promoting technological innovation. They adjust to enterprises’ production technologies through regulations and market tools [18]. It can be found that European countries have significantly promoted the development and utilization of green technologies by using environmental taxation, laying the foundation for sustainable development while reducing environmental pollution.

It can be observed that in the first two years of implementing the Environmental Protection Tax Law, namely 2018 and 2019, the “Porter Hypothesis” effect was not significant. This implies that the Environmental Protection Tax Law failed to promote enterprise development, although it played a positive role in environmental protection. However, owing to its unfavorable impact on enterprise development, this environmental protection effect is unlikely to be sustainable. China implemented epidemic prevention and control measures from early 2020 to the end of 2022. During this period, the regulatory function of the Environmental Protection Tax Law remained uncertain. Therefore, it is necessary to explore this issue, as it will not only help reveal the operational mechanisms of the Environmental Protection Tax Law during these three years but also identify existing problems. Furthermore, the valuable experience gained during the pandemic can serve as a guide for future revisions and adjustments in the post-pandemic period, thereby enabling the Environmental Protection Tax Law to achieve its environmental protection and economic goals at an earlier stage.

It can be seen from the existing research that there has been much discussion on whether environmental taxes can promote environmental performance, but the existence of the pandemic has seriously affected the effectiveness of environmental taxes, and this aspect has been less discussed. At the same time, the discussion on fairness and rationality in the process of environmental protection tax collection is also insufficient, and the environmental regulation effect of the environmental protection tax can only be guaranteed based on the effective implementation of policies. In addition, there is dearth of studies on the mechanism of Porter’s hypothesis in the Chinese context along with insufficient discussion on the differences between strong and weak Porter’s hypothesis in influencing the technological innovation of Chinese enterprises.

China’s environmental quality is of great importance as it is an important part of the world. After decades of development, China’s economy has made significant progress; however, economic growth has been achieved at the expense of environmental resources. As the world faces an increasingly severe ecological situation, China needs to take measures to change its traditional industrial and technological structures and vigorously develop an environmentally friendly economy. The proposal of an environmental protection tax law is important for China to cope with environmental crises and achieve sustainable economic and environmental development. This experience offers valuable insights for the global community on usage of environmental regulations to foster the development of green technologies and establish new models of industrial growth. China’s market-oriented approach allows for governmental intervention to steer corporate behavior, whereas Western nations often rely on market incentives to promote green practices, resulting in some differences. Therefore, based on China’s experience, jointly exploring enterprises’ green development paths have important theoretical and practical value.

It aims to verify the Porter Hypothesis effect of environmental taxes and identify existing issues in the current implementation of China’s Environmental Protection Tax Law. Maximizing the law’s potential for environmental protection and economic drive offers valuable lessons for countries worldwide to enhance green behavior through environmental regulations, ultimately fostering sustainable development.

The article structure is as follows: First, the research background is introduced in the Introduction section, and the shortcomings of the existing research and the significance of this research are pointed out through a review of the relevant literature. Second, with the help of literature analysis, the research hypotheses are proposed, and the research model is pointed out. Third, the questionnaire was introduced through the design of the research questionnaire, the questionnaire data collection process was expounded, and the research model was tested using the questionnaire data. Fourth, based on the research model test results, countermeasures and suggestions for the problems existing in the implementation process of environmental protection tax laws are presented. Finally, limitations and future research directions are discussed.

## 2 Research model design of the “Porter Hypothesis” effect

### 2.1 Analysis of the “Strong Porter Hypothesis” effect

The “Strong Porter Hypothesis” effect of the Environmental Protection Tax Law refers to the implementation of the law not only for enhancing the green innovation capacity of enterprises but also improving their overall technological innovation capability. The growth in this overall technological innovation capability inevitably enhances an enterprise’s competitive advantage [19]. The “Strong Porter Hypothesis” effect is the ultimate goal of the implementation of the Environmental Protection Tax Law in China. This not only intensifies environmental protection efforts, improving the ecological environment, but also enhances the competitive advantage of enterprises and boosts their operational performance. Fairness and rationality are foundational principles of effective taxation systems. Only by embodying these principles within the Environmental Protection Tax Law can the “Strong Porter Hypothesis” effect be achieved [20]. Fairness refers to the implementation and enforcement of the “Environmental Protection Tax Law” equally to all heavily polluting enterprises. This entails treating all such enterprises equally in terms of tax amounts, taxation procedures, information-sharing, preferential subsidies, and avoidance of favoritism. There should be no discrimination in favor of or against any company. Rationality, on the other hand, refers to the implementation and enforcement of the “Environmental Protection Tax Law” in accordance with predetermined procedures and requirements. This includes maintaining compliance and rationality in measuring pollutant emissions, calculating tax amounts, implementing information disclosure systems, setting tax rates for different industries and regions, and ensuring a certain level of implementation quality [21].

Based on this discussion, the following research hypotheses can be proposed:

H11: The fairness of the Environmental Protection Tax Law enhances the competitive advantage of heavily polluting enterprises.
H12: The rationality of the Environmental Protection Tax Law can enhance the competitive advantage of heavily polluting enterprises.

### 2.2 Analysis of the “Weak Porter Hypothesis” effect

#### 2.2.1 Analysis of the promotional effect of tax fairness on the green technology innovation capability of heavily polluting enterprises

Tax fairness is an important goal in the implementation and improvement of the “Environmental Protection Tax Law” because it is only through fairness that the law can effectively foster the promotion of green innovation capability among heavily polluting enterprises. The implementation time of the Environmental Protection Tax Law was relatively short, and unfair factors still exist in many taxation aspects that must be gradually curbed [22]. As a highly anticipated environmental regulation, the promotion of competitive advantages for heavily polluting enterprises through the “Environmental Protection Tax Law” is a gradual process that requires the achievement of green technology innovation first before overall technological innovation can be expected. This is because green technology innovation is only one element of overall technological innovation and not its entirety [23]. According to environmental or green production standards, when heavily polluting enterprises reach the level of green technology innovation, it does not necessarily mean that they have achieved the overall level of technological innovation. Only through overall technological innovation can an enterprise’s competitive advantage be enhanced. Green technology innovation capabilities generally consist of three basic elements: green process innovation, green product innovation, and waste treatment innovation. Therefore, tax fairness in the implementation of the “Environmental Protection Tax Law” promotes these three elements at the micro-level to enhance green technology innovation capability [24]. In particular, waste treatment innovation capability is a core element of the green technology innovation capability of heavily polluting enterprises.

Based on this discussion, the following research hypotheses are proposed:

H21: Tax fairness in implementing the “Environmental Protection Tax Law” enhances the green process innovation capabilities of heavily polluting enterprises.
H22: Tax fairness in implementing the “Environmental Protection Tax Law” enhances the green product innovation capabilities of heavily polluting enterprises.
H23: Tax fairness in implementing the “Environmental Protection Tax Law” enhances the waste treatment innovation capabilities of heavily polluting enterprises.

#### 2.2.2 Analysis of the promotional effect of tax rationality on the green technology innovation capability of heavily polluting enterprises

Tax rationality is another important objective of implementing and improving the Environmental Protection Tax Law. The Environmental Protection Tax Law can effectively promote the green technology innovation capabilities of heavily polluting enterprises only when it exhibits reasonable characteristics in terms of emission measurement, tax calculation, information disclosure, tax rate setting, and coordination between environmental protection and tax departments [25]. The Environmental Protection Tax Law is the result of the “fee-to-tax” reform, and the tax administration department had no experience in tax collection before 2018. After only two years of implementation, it faced three years of epidemic prevention and control, making it difficult for tax administration departments to achieve complete rationality in the tax collection process. However, many unresolved issues hinder rationality [26]. The promotion of green technology innovation of heavily polluting enterprises under the Environmental Protection Tax Law is a gradual process. The more rational the tax collection behavior, the higher the enthusiasm of enterprises to engage in green technology innovation, leading to increased investment in green technology research and more significant effects of green technology innovation [27].

Based on the above arguments, the following research hypotheses can be proposed:

H31: Tax rationality of the Environmental Protection Tax Law can enhance the green process innovation capabilities of heavily polluting enterprises.
H32: Tax rationality of the Environmental Protection Tax Law can enhance the green product innovation capabilities of heavily polluting enterprises.
H33: Tax rationality of the Environmental Protection Tax Law can enhance the innovative waste treatment capabilities of heavily polluting enterprises.

### 2.3 Analysis of the transition from “Weak Porter Hypothesis” to “Strong Porter Hypothesis” effect

The basic characteristic of the “Weak Porter Hypothesis” effect is that under the promotion of the “Environmental Protection Tax Law,” the green technology innovation capability of heavily polluting enterprises significantly increases. On the other hand, the basic characteristic of the “Strong Porter Hypothesis” effect is that, under the promotion of the “Environmental Protection Tax Law,” the overall technological innovation capability of heavily polluting enterprises significantly increases, or it is reflected as a significant improvement in their competitive advantage. Worldwide, whether in developed or developing nations, the realization process of any environmental regulation’s “Porter Hypothesis” effect follows a progressive transition from the “Weak Porter Hypothesis” to the “Strong Porter Hypothesis” effect. China’s “Environmental Protection Tax Law” is no exception [28]. Since the beginning of the reform and opening-up policy, China’s economic output has grown rapidly. However, it has also left behind a significant number of environmental pollution issues, with the impact of heavily polluting enterprises being particularly prominent. In this context, even with the vigorous implementation of the “Environmental Protection Tax Law,” it is challenging to achieve the “Strong Porter Hypothesis” effect in the short term. For heavily polluting enterprises, only when their green technology innovation capability reaches a certain level can it drive the growth of overall technological innovation [29]. Thus, it can be argued that the realization of the “Strong Porter Hypothesis” effect is only possible under the promotion of the “Weak Porter Hypothesis” effect.

Based on these arguments, the following research hypotheses can be proposed:

H41: Green technological process innovation capability of heavily polluting enterprises can enhance their competitive advantage.
H42: Green product innovation capability of heavily polluting enterprises can enhance their competitive advantage.
H43: The innovative waste treatment capabilities of heavily polluting enterprises can enhance their competitive advantages.

### 2.4 Research model design of the “Porter Hypothesis” effect

**Fig 1:**
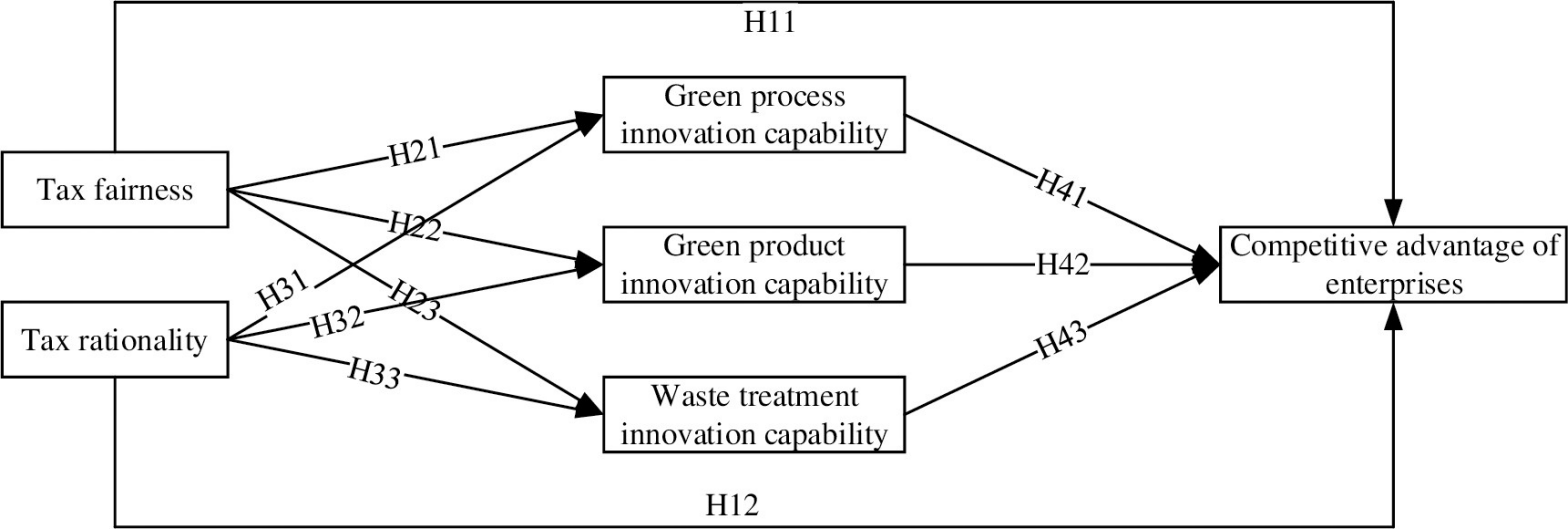
Research model.

This study examines research hypotheses using structural equation modeling (SEM). According to SEM theory, a research model can be constructed based on the induction of research hypotheses, as illustrated in Fig 1. The research model comprised two independent variables, three mediating variables, one dependent variable, and 11 causal paths.

## 3 Examination of the research model on the “Porter Hypothesis” effect

### 3.1 Questionnaire design

#### 3.1.1 Questionnaire design on the regulation of the “Environmental Protection Tax Law”

Tax fairness is an ideal demand of enterprises concerning tax levying, manifested as the equality of all enterprises with respect to tax laws, whereby every business is treated equally. Drawing from relevant studies, the questionnaire design on the fairness of taxation in the context of “Environmental Protection Tax Law” includes the following items: 1) Fairness of tax outcomes, which refers to fairness in environmental protection tax collection from all enterprises within the industry; 2) Fairness of tax procedures, which indicates the fairness of tax collection actions by tax personnel for all enterprises within the industry; 3) Fairness of tax information, which refers to fairness of tax-related information accessible to the taxpayer enterprises; 4) Fairness of tax incentives, which is the fairness of preferential reductions or exemptions of environmental protection tax for any industry and enterprises within the industry [30].

Tax rationality is rightful expectation of taxpayer enterprises regarding taxation, reflecting the need for tax authorities to adopt scientific and effective tax measures to ensure the quality of taxation. Drawing from relevant studies, the questionnaire design on the tax rationality in the context of “Environmental Protection Tax Law” includes the following items: 1) Rationality of tax rate setting, which refers to the rationality of tax rate levels set by tax authorities in the local region and industry; 2) Rationality of emission measurement, which indicates rationality of measurement methods employed by environmental protection department for wastewater, emissions, and waste discharge by enterprises; 3) Rationality of tax calculation, which refers to the rationality of tax calculation for taxable amounts by tax authorities; 4) Rationality of information disclosure, indicating rationality of tax-related information disclosure by tax authorities in terms of content, timing, and methods [31].

#### 3.1.2 Questionnaire design on the green technology innovation capability of heavily polluting enterprises

Drawing on relevant research, the questionnaire design on the green process innovation capability of heavily polluting enterprises includes the following items: 1) Innovation in green procurement, which refers to the continuous innovation in the procurement of raw materials by enterprises to support low-carbon environmental practices. 2) Innovation in green production, indicating continuous innovation in the production processes by enterprises to reinforce low-carbon environmental protection. 3) Innovation in green equipment, which refers to continuous innovation in the utilization and modification of production equipment by enterprises for low-carbon environmental protection. 4) Innovation in green sales, indicating continuous innovation in the design, implementation, and development of product sales channels by enterprises in terms of low-carbon environmental protection [32].

Drawing on relevant research, the questionnaire design on the green product innovation capability of heavily polluting enterprises includes the following items: 1) Innovation in green performance, which indicates the extent to which a company’s products contribute to environmental protection and energy conservation through improved performance. 2) Innovation in green environmental protection, indicating the extent to which a company’s products contribute to environmental protection during consumption or use. 3) Innovation in green energy conservation, which refers to the extent to which a company’s products contribute to energy conservation during consumption or use. 4) Innovation in green concepts, indicating the extent to which the presence of a company’s products can guide or deepen the public’s awareness of green concepts [33].

Drawing on relevant research, the questionnaire design on waste management innovation capability in heavily polluting enterprises includes the following items: 1) Innovation in water pollution treatment, indicating innovation capability in the treatment of water pollutants. 2) Innovation in air pollution treatment, referring to innovation capability in the treatment of air pollutants. 3) Innovation in solid waste treatment, which indicates innovation capabilities in the treatment of solid waste. 4) Innovation in noise control, referring to innovation capability in noise control methods or effectiveness [34].

#### 3.1.3 Questionnaire design on the competitive advantage of heavily polluting enterprises

Drawing on relevant research, the questionnaire design on the competitive advantage of heavily polluting enterprises includes the following items: 1) Industry competitive advantage, implying strong competitive advantage of enterprises within the industry. 2) Regional competitive advantage, which refers to strong competitive advantage of enterprises within a region. 3) Domestic competitive advantage, which indicates enterprises’ strong competitive advantage in the domestic market. 4) International competitive advantage, indicating strong competitive advantage of enterprises in the international market [35].

### 3.2 Sample survey

Heavily polluting industries refer to industries causing severe environmental pollution during the production and application of products. On September 14, 2010, the Ministry of Environmental Protection announced the “Guidelines for Environmental Information Disclosure of Listed Companies,” identifying 16 industries, including thermal power, steel, cement, coal, chemical, electrolytic aluminum, metallurgy, petrochemical, brewing, building materials, pharmaceuticals, papermaking, fermentation, tanning, textiles, and mining, as heavily polluting industries.

This study used enterprises from heavily polluting industries in China as samples and conducted a survey using a 7-point scale. The survey was conducted from December 15th to 31st, 2022. A total of 456 samples were obtained, and after eliminating invalid responses with missing items and repeated information, 400 valid samples were shortlisted. In this study, all six factors–tax fairness, tax rationality, green process innovation capability, green product innovation capability, waste treatment innovation capability, and corporate competitive advantage–were measured on a 7-point scale. Survey participants were senior executives of heavily polluting enterprises. They not only possess the ability to assess the implementation levels of tax rationality and tax fairness but also have a clear understanding of the achievement level of their enterprises’ in terms of green process, green product, and waste treatment innovation capabilities as well as their company’s leading position in the industry, region, domestic, and international markets. Prior to the survey, the respondents were informed that the evaluation pertained only to the period of epidemic prevention and control, spanning from early 2020 to the end of 2022.

To ensure the scientific validity of the survey, a preliminary investigation of the enterprises was conducted before the survey to ensure that they belonged to the category of heavily polluting industries. To further ensure the scientific validity of enterprise evaluation, the survey subjects were determined through government and market enterprise catalogs, and accordingly senior executives of heavily polluting enterprises were selected. Because the survey targets were enterprise executives, communication with them was arranged in advance to determine the mode of data collection, such as telephone, online communication tools, or on-site communication with team members. Before the survey, the participants were briefed on the relevant information in the survey questionnaire to avoid misunderstandings. As this was a study on China’s Environmental Protection Tax Law, the research subjects were senior management personnel of heavily polluting Chinese enterprises. Mandarin Chinese was used as the language to facilitate the understanding of the survey content by the subjects. The entire survey process was led by the author and completed with the assistance of team members.

The characteristics of the sample are presented in Table 1, where the number of employees and enterprise profits represent the values in 2021. By analyzing the characteristics of the sample data, they can be preliminarily summarized and sorted to determine whether the research object is reasonable and the investigation data are trustworthy.

**Table 1:** Characteristics of the samples.

Attributes	Type	Share	Percentage%	Attributes	Type	Share	Percentage%
Regional Distribution	North China	64	16	Employee Distribution	< = 500 employees	120	30
	South China	72	18		501–1000 employees	88	22
	East China	76	19		1001~1500 employees	60	15
	Southeast China	60	15		1501~2000 employees	48	12
	Northeast China	38	9.5		2001~2500 employees	36	9
	Northwest China	46	11.5		2501~3000 employees	28	7
	Southwest China	44	11		> = 3001 employees	20	5
Industry Distribution	Thermal Power	18	4.5	Company Age Distribution	< = 5 years	116	29
	Iron and Steel	16	4		6~10 years	76	19
	Cement	12	3		11~15 years	62	15.5
	Coal	22	5.5		16~20 years	50	12.5
	Chemical Industry	32	8		21~25 years	44	11
	Aluminum Smelting	8	2		25~30 years	36	9
	Metallurgy	28	7		> = 31 years	16	4
	Petrochemical	24	6	Company Profit Distribution	< = 5 million yuan	112	28
	Brewing	20	5		5–10 million yuan	100	25
	Building Materials	44	11		10–15 million yuan	76	19
	Pharmaceutical	28	7		15–20 million yuan	44	11
	Papermaking	18	4.5		20–25 million yuan	30	7.5
	Fermentation	16	4		25–30 million yuan	22	5.5
	Leathermaking	14	3.5		> = 30 million yuan	16	4
	Textile	28	7	Enterprise attribute distribution	State-owned enterprises	162	40.5
	Mining	20	5		Private enterprises	206	51.5
	Other	52	13		Foreign-funded enterprises	32	8

The research model consisted of six factors, with each factor comprising four items, thus totaling to 24 questionnaire items. The statistical characteristics of the sample data based on 400 valid samples are presented in Table 2.

**Table 2:** Statistical characteristics of sample data.

Element name	Sample size	Maximum	Minimum	Mode	Mean	Variance
Tax fairness	400	7	1	3	3.12	0.12
Tax rationality	400	7	1	2	2.36	0.09
Green process innovation capability	400	7	1	2	2.28	0.10
Green product innovation capability	400	7	1	3	3.17	0.16
Waste treatment innovation capability	400	7	1	4	3.95	0.13
Competitive advantage of enterprises	400	7	1	3	3.03	0.16

### 3.3 Reliability test

Questionnaire reliability refers to the consistency and reliability of the measurement results obtained through the questionnaire. When conducting surveys using questionnaires, errors are inevitable, leading to discrepancies between the true and measured values. The larger the error, the lower is the reliability. In this study, based on a sample of 400 respondents, the reliability test was conducted using Stata 15.0 software. The reliability test results for “Regulation of Environmental Protection Tax Law,” “Green Technology Innovation Capability,” and “Enterprise Competitive Advantage” are presented in Tables 3–5, respectively. As can be inferred from the tables, each factor demonstrated good reliability.

**Table 3:** Reliability test results of "Regulation of Environmental Protection Tax Law" factors.

Factor system	Corrected Item-Total Correlation	Alpha If Item Deleted	Coefficient Alpha Cronbach’s
Tax fairness			0.8989
Fairness of tax results	0.6231	0.7828	
Fairness of tax procedures	0.5360	0.7769	
Fairness of tax information	0.5512	0.7676	
Fairness of tax incentives	0.6340	0.8715	
Tax rationality			0.7899
Rationality of tax rates	0.7122	0.9310	
Rationality of emission measurements	0.6850	0.8819	
Rationality of tax calculation	0.5328	0.7018	
Rationality of information disclosure	0.6281	0.7966	

**Table 4:** Reliability test results of "Green technology innovation capability" factors.

Factor system	Corrected Item-Total Correlation	Alpha If Item Deleted	Coefficient Alpha Cronbach’s
Green process innovation capability			0.9120
Green procurement innovation	0.5129	0.7860	
Green production innovation	0.6102	0.8218	
Green equipment innovation	0.6343	0.8761	
Green sales innovation	0.5617	0.7719	
Green product innovation capability			0.8891
Green performance innovation	0.5219	0.7818	
Green environmental protection innovation	0.6710	0.8341	
Green energy-saving innovation	0.6561	0.8127	
Green process innovation	0.5535	0.7652	
Waste treatment innovation capability			0.8219
Water pollutant treatment innovation	0.5229	0.7819	
Air pollutant treatment innovation	0.5062	0.7528	
Solid waste treatment innovation	0.6319	0.8365	
Noise control innovation	0.6615	0.8278	

**Table 5:** Reliability test result of "Competitive advantage of enterprises" factors.

Factor system	Corrected Item-Total Correlation	Alpha If Item Deleted	Coefficient Alpha Cronbach’s
Competitive advantage of enterprises			0.8367
Competitive advantage of industry	0.6098	0.7910	
Competitive advantage of region	0.5179	0.7719	
Competitive advantage in domestic market	0.4466	0.7279	
Competitive advantage in international market	0.6390	0.8217	

### 3.4 Model verification

Based on the reliability test, the research model was examined using Stata 15.0 and Lisrel 8.7 software. The results of the model testing are presented in Table 6.

**Table 6:** Results of model verification.

Index	Hypothesis	Hypothesis content	Path coefficient	T-value	Test result
1	H11	Tax fairness → Competitive advantage of enterprises	0.37	3.85	Accepted
2	H12	Tax rationality → Competitive advantage of enterprises	0.11	1.72	Rejected
3	H21	Tax fairness → Green process innovation capability	0.30	4.33	Accepted
4	H22	Tax fairness → Green product innovation capability	0.27	2.91	Accepted
5	H23	Tax fairness → Waste treatment innovation capability	0.26	5.18	Accepted
6	H31	Tax rationality → Green process innovation capability	0.13	1.07	Rejected
7	H32	Tax rationality → Green product innovation capability	0.09	1.11	Rejected
8	H33	Tax rationality → Waste treatment innovation capability	0.30	3.95	Accepted
9	H41	Green process innovation capability → Competitive advantage of enterprises	0.12	1.78	Rejected
10	H42	Green product innovation capability → Competitive advantage of enterprises	0.34	2.99	Accepted
11	H43	Waste treatment innovation capability → Competitive advantage of enterprises	0.33	3.78	Accepted

Furthermore, the goodness-of-fit indices of the research model are presented in Table 7. It can be observed that the model testing results indicate a good fit, suggesting no need for modifications to the research model design.

**Table 7:** Goodness-of-fit indices for model verification.

Fit index type	Fit index name	Fit index value
Absolute fit index	*χ*^2^/*df*	1.921
	*GFI*	0.911
	*AGFI*	0.920
	*RMSEA*	0.034
Relative fit index	*NFI*	0.987
	*TLI*	0.926
	*CFI*	0.933
Information criteria index	*AIC*	98.16
	*CAIC*	125.34
	*ECVI*	0.446

**Fig 2:**
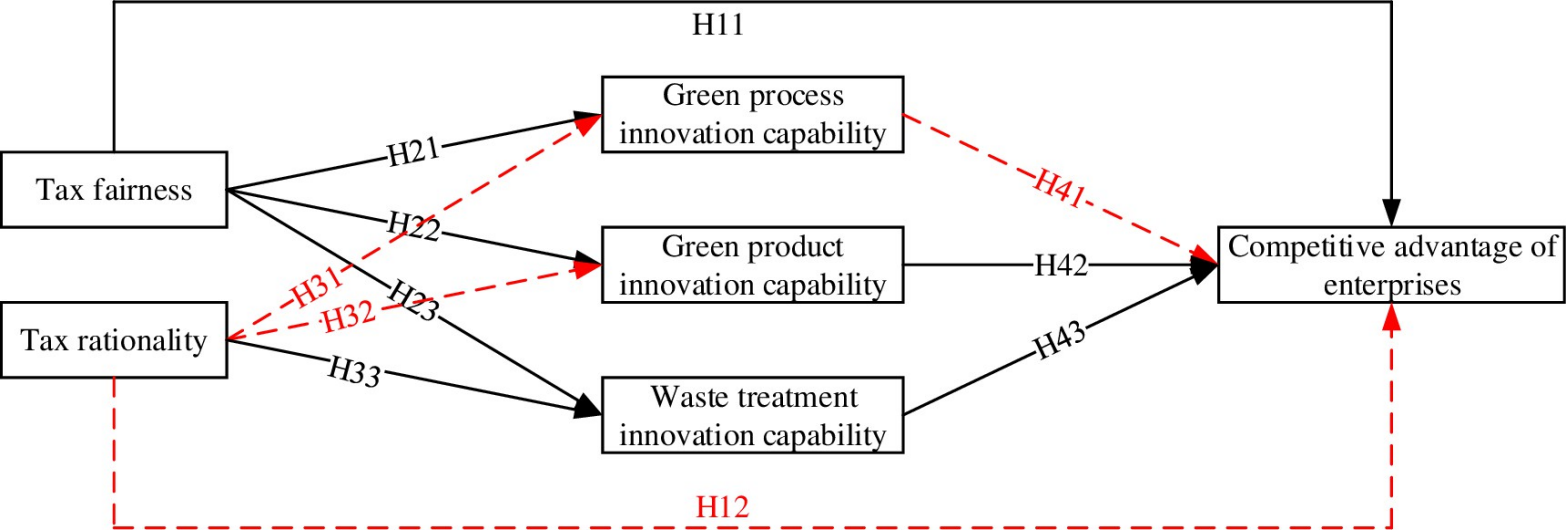
Results of model verification.

The results of the model testing according to the model design are illustrated in Fig 2.

Based on the test results, the following conclusions can be drawn: 1) Hypothesis H11 was supported, whereas Hypothesis H12 was not supported, indicating that the “Porter Hypothesis” effect was only partially realized. Specifically, it is achieved in terms of tax fairness, but not tax rationality. 2) Hypotheses H31 and H32 were not supported, while H21, H22, H23, and H33 were supported, suggesting that the “Weak Porter Hypothesis” effect is only partially realized. It is achieved in terms of tax fairness but not significantly in terms of tax rationality. 3) Hypothesis H41 was not supported, whereas H42 and H43 were supported, indicating that the path of transformation from the “Weak Porter Hypothesis” to the “Strong Porter Hypothesis” is only partially realized among heavily polluting enterprises in China.

## 4 Discussion

Using structural equation modelling on sample data from heavily polluting enterprises, this study empirically tested the Porter Hypothesis effect of Environmental Protection Tax Law during the period of strict epidemic prevention and control. According to the model test results, some research hypotheses have not been supported, indicating that during the period of strict control, the environmental regulation function of China’s Environmental Protection Tax Law could not be realized; Porter’s hypothesis did not fully materialize in the Chinese context, and its promotion of enterprises’ green technology innovation capabilities fell short, thereby affecting their market competitiveness.

Although Porter’s hypothesis has been proven to play a role in China’s management context, which is conducive to the realization of enterprises’ technological innovation capabilities [36], the effectiveness of environmental regulation under sudden crises has not been thoroughly discussed. Regional differences cause heterogeneity in the implementation of environmental protection tax laws [37], but the fundamental problem still lies in whether the environmental tax is fair in its implementation process and whether the environmental regulations are reasonable, which ultimately influences the technological innovation of enterprises. Previous studies have found that environmental taxes significantly stimulate enterprises’ green technology innovation behavior in the rapid development stage of the market economy [38], and the Porter Hypothesis effect of the Environmental Protection Tax Law can reach an ideal level under favorable market conditions [39–41]. As environmental policies evolve in various regions, the development of environmental taxes, similar to those in European Union countries, will gradually converge [42]. This implies the need for continuous optimization and adjustment of the Environmental Protection Tax Law to create a foundation for sustainable development [43]. Environmental taxes need to be adjusted based on the reality to ensure the effectiveness of environmental regulations; otherwise, it is detrimental to both environmental protection and economic development, and may even lead to social crises [44]. Through the study of the Porter Hypothesis effect of Environmental Protection Tax Law during the period of strict control, the defects of environmental regulation policies can be better discovered, the weak Porter Hypothesis effect can be promoted to the strong Porter Hypothesis effect, and the mutual development of technological innovation and environmental protection can be achieved.

The results of the model test show that the strong Porter effect of the Environmental Protection Tax Law is insufficient. Hypothesis H12 failed to pass the model verification, indicating that the tax rationality of the Environmental Protection Tax Law was insufficient. It fails to comply with predetermined procedures and requirements in the process of environmental tax collection, and there are deficiencies at the standard formulation level. For enterprises, the lack of a unified tax standard makes it difficult for them to formulate their own strategic planning according to environmental taxation, which restricts their investment in technological innovation and ultimately hampers their competitive advantage in the market. Tax fairness is a premise of environmental regulations. Enterprises can fulfill their environmental tax obligations only by ensuring fairness.

In the weak Porter Hypothesis effect, the promotion effect of tax fairness on green technology innovation has been verified, which once again shows that under a fair tax environment, enterprises are motivated to carry out green technology innovation. Hypotheses H31 and H32 were not supported, indicating that tax rationality is a key factor restricting the weak Porter Hypothesis effect. Rationality should not only be enforced at the system level, but should also be reflected in the implementation process of environmental protection tax. As the differences in production and operation activities between enterprises are objective and the differences in the regional economy are more significant, it is necessary to adjust the implementation of the Environmental Protection Tax Law according to the actual situation, ensure the regulation of the Environmental Protection Tax Law, and ensure the rationality of taxation to effectively stimulate the green technology innovation potential of enterprises.

The transformation from the weak Porter Hypothesis effect to the strong Porter Hypothesis effect is the key objective of the Environmental Protection Tax Law, and it is also a gradual process. According to the model test results, it is evident that the transition from the weak Porter Hypothesis effect to the strong Porter Hypothesis effect progressed. However, shortcomings remain, indicating that the green technology innovation capability of heavily polluting enterprises has not yet been fully realized. Traditional production processes still prevail, which is not only detrimental to the transformation and upgrading of industrial structure but also hinders the shaping of competitive advantages for enterprises. Therefore, the key focus of environmental regulation for heavily polluting enterprises in China should be on guiding the green transformation and upgrading of production processes, utilizing green development to shape market advantages, and further promoting the transition process from a weak to a strong Porter Hypothesis effect.

## 5 Conclusions and policy implications

### 5.1 Research conclusions

This study uses taxation fairness and rationality as independent variables; green technology, green product, and waste treatment innovation capabilities as mediating variables; and enterprise competitive advantage as the dependent variable. It constructs a structural equation model containing 11 causal paths to verify the Porter Hypothesis effect of the Environmental Protection Tax Law and reveals the mechanism of action between strong and weak Porter Hypothesis effects. According to the results of the model test, the Porter Hypothesis effect of the Environmental Protection Tax Law has partially played a role, but deficiencies exist in the transition path from a weak to strong Porter Hypothesis effect. Additionally, both the strong and weak Porter Hypothesis effects of the Environmental Protection Tax Law have shortcomings, resulting in the incomplete realization of green technology innovation capability in heavily polluting enterprises and the failure to establish a more comprehensive competitive advantage for enterprises.

In the model test results, the path coefficient was lower than 0.2 and the T-value was lower than 2.0, indicating that the model hypothesis, as represented by the path, was rejected because the path coefficient was not statistically significant. In the analysis of the strong Porter Hypothesis effect, hypothesis H11 (Tax fairness → Competitive advantage of enterprises, path coefficient 0.37 > 0.2, T value 3.85 > 2.0) was supported, indicating that taxation fairness contributes to the enhancement of enterprise competitive advantage. Taxation fairness is the foundation of an enterprise’s operations. Any heavily polluting enterprise facing pressure due to environmental protection taxes must act to promote the transformation and upgrading of enterprise technology. However, H12 (Tax rationality → Competitive advantage of enterprises, path coefficient 0.11 < 0.2, T value 1.72 < 2.0) was not supported. This suggests that the collection of environmental protection taxes during the control period was not rational and could not help enterprises establish a competitive advantage. Faced with unreasonable environmental tax collection, enterprises adopted a passive attitude and missed opportunities to establish a competitive advantage.

In the analysis of the weak Porter Hypothesis effect, hypotheses H21 (Tax fairness → Green process innovation capability, path coefficient 0.30> 0.2, T value 4.33> 2.0), H22 (Tax fairness → Green product innovation capability, path coefficient 0.27> 0.2, T value 2.91> 2.0), and H23 (Tax fairness → Waste treatment innovation capability, path coefficient 0.26> 0.2, T value 5.18> 2.0) were supported. This shows that tax fairness significantly promotes the green technology innovation ability of enterprises; particularly at the micro level because, regardless of the nature of enterprises, they must face the pressure of environmental tax, and to better cope with environmental regulations, enterprises must invest resources in green technology research and development to reduce the pressure of environmental tax. Hypotheses H31 (Tax rationality → Green process innovation capability, path coefficient 0.13 < 0.2, T value 1.07 < 2.0) and H32 (Tax rationality → Green product innovation capability, path coefficient 0.09 < 0.2, T value 1.11 < 2.0) were not supported, whereas hypothesis H33 (Tax rationality → Waste treatment innovation capability, path coefficient 0.30 > 0.2, T value 3.95 > 2.0) was supported. This indicates shortcomings in the regulation of taxation rationality in the production process of environmental protection tax collection. Waste treatment has been emphasized as an easily observable indicator. Irrationality in the taxation process easily leads to opportunistic behavior, causing enterprises to prefer investment in waste treatment while neglecting green development in the fields of technology and product innovation.

In the analysis of the transition from the weak to strong Porter Hypothesis, hypothesis H41 (Green process innovation capability → Competitive advantage of enterprises, path coefficient 0.12 < 0.2, T value 1.78 < 2.0) was not supported. However, hypotheses H42 (Green product innovation capability → Competitive advantage of enterprises, path coefficient 0.34 > 0.2, T value 2.99 > 2.0) and H43 (Waste treatment innovation capability → Competitive advantage of enterprises, path coefficient 0.33 > 0.2, T value 3.78 > 2.0) were supported. This indicates that green technology innovation capability does not effectively promote the establishment of enterprise competitive advantage. This can be attributed to the complexity of the technology involved in the process, which requires more investment, and the fact that it takes a considerable amount of time for companies to recover their R&D costs after completing green technology transformations. In contrast, green product innovation and waste treatment require relatively fewer resources, allowing companies to gain better market recognition by promoting green products.

The Porter Hypothesis effect of the Environmental Protection Tax Law during the period of strict control has been partially realized and the promotion effect of the green technology innovation ability of heavily polluting enterprises has been partially verified, which is conducive to shaping the competitive advantage of enterprises. However, according to the results of the model test, the rationality of taxation is the biggest factor restricting the effect of Environmental Protection Tax Law during the control period. Faced with a lack of reasonable taxation, entrepreneurs are compelled to adopt negative measures to cope with the regulation of environmental taxation, utilizing pollution management innovation, which is easier to regulate, to reduce business costs and gain opportunity cost advantages. This seriously undermines the effective implementation of the Environmental Protection Tax Law, harming both the green transformation and upgrading of industrial structures, and the sustainable development of the ecological environment. Strengthening taxation rationality will be a key focus of future work to better leverage the Porter Hypothesis effect of the Environmental Protection Tax Law and achieve synchronized and healthy economic and environmental development. This also provides a reference for the comprehensive implementation of environmental protection tax laws worldwide, ensuring that both the fairness and rationality of tax laws are essential for their respective effectiveness.

### 5.2 Policy implications

In the post-pandemic era, it is essential not only to deepen the understanding of the “Porter Hypothesis” effect under the regulation of the “Environmental Protection Tax Law” but also to make improvements to the regulation itself.

Firstly, during the three years of epidemic prevention and control, the “Porter Hypothesis” effect of the Environmental Protection Tax Law has not shown significant improvement, indicating a lack of preferment for green technology innovation and overall technological innovation in heavily polluting enterprises. In other words, even five years after the implementation of the Environmental Protection Tax Law, the expected goals have not been achieved for heavily polluting industries. Therefore, for future economic development, the Chinese government and society should continue to attach great importance to promoting the role of the “Environmental Protection Tax Law” in clean production, energy conservation, emission reduction, and green transformation of heavily polluting enterprises. Efforts should be made to strengthen the implementation of the Environmental Protection Tax Law, intensify the governance of heavily polluting enterprises, and encourage them to increase investments in green environmental protection. Simultaneously, every effort should be made to advance green technological innovations. Thereby, while achieving the ecological and environmental protection goals, the financial performance of these enterprises can also be enhanced.

Second, it is crucial to enhance the rationality of tax collection under the Environmental Protection Tax Law to fully realize the “Porter Hypothesis.” According to the verification results, the “Weak Porter Hypothesis” and the “Strong Porter Hypothesis” effects have been achieved in terms of tax fairness, but not in terms of tax rationality. This indicates that during the three-year period of pandemic prevention and control, the “Porter Hypothesis” effect was only partially realized for heavily polluting enterprises, with the function of tax fairness being effectively exerted, while the function of tax rationality was constrained. Therefore, in future environmental tax reforms, tax authorities should not only improve the rationality of tax rate setting, emission measurement methods, and tax calculations for heavily polluting industries, but also deepen the system of tax information disclosure and enhance the rationality of information disclosure.

Third, under the Environmental Protection Tax Law, heavily polluting enterprises should increase their investments in green technological and product innovation. During the three years of pandemic prevention and control, heavily polluting enterprises generally reduced their investments in green process innovation and green product innovation because of the decline in economic performance. This has been a significant factor that has led to a decrease in the effectiveness of the “Environmental Protection Tax Law,” subsequently damaging the competitive advantage of these enterprises. Therefore, during the coexistence with the virus, heavily polluting enterprises should gradually increase their investment in green technology and green product innovation, striving to restore their pre-epidemic levels as soon as possible. This will not only improve their capabilities in green technology and green product innovation but also enhance their competitive advantages. In the early stages of coexistence with the virus, if heavily polluting enterprises quickly increase their investment in green technology and product innovation, they can gain market control initiatives at the earliest. Conversely, if actions are delayed, they may miss market opportunities.

Lastly, it is essential to leverage the waste treatment innovation capability as a bridge during the transition from the “Weak Porter Hypothesis” effect to the “Strong Porter Hypothesis” effect for heavily polluting enterprises. During the three years of epidemic prevention and control, the “Environmental Protection Tax Law” has shown a significant promoting effect on the innovative waste treatment capabilities of heavily polluting enterprises. Innovative waste treatment capabilities also significantly contribute to the competitive advantage of these enterprises. This indicates that the environmental protection function of the “Environmental Protection Tax Law” still exists and has not completely faded away. It also plays a crucial role in the supervision of waste discharge. Therefore, during the coexistence with the virus, Chinese government departments, such as tax and environmental protection, along with heavily polluting enterprises, should learn from the experience of how the regulation of the “Environmental Protection Tax Law” promoted waste treatment innovation during the three years of epidemic prevention and control. By doing so, they can further enhance their regulatory roles and achieve greater effectiveness.

### 5.3 Limitations and future prospects

Based on structural equation theory, this study explores the environmental regulation effect of the Environmental Protection Tax Law under the Porter Hypothesis and uses heavily polluting enterprises as samples to analyze the mechanism of environmental tax on green technology innovation and corporate competitive advantage. Limited by the author’s knowledge system and specialty, the study has the following limitations: 1) The study sample only includes heavily polluting enterprises, and the effect of environmental protection tax on other types of enterprises is not discussed. 2) The study sample is confined to China and ignores other countries, which is not conducive to analyzing the effectiveness of environmental regulation under different market mechanisms. 3) Insufficient discussions on enterprises’ competitive advantages. In the context of the global pursuit of green development, the green behavior of enterprises can be regarded as the embodiment of market competitiveness, but this factor is not considered in the research process.

To further optimize the research, future research direction can start from the field of improvement of the green industry chain through green technology innovation behavior of enterprises, under the background of environmental regulation. The environmental regulation effect of the Environmental Protection Tax Law has not been fully realized, not only because of the tax law itself but also because of the lack of industrial chain’s recognition and support for green technology.

## Supporting information

S1 Data (DOCX)
