# Supplementary material for: A study on the "Porter Hypothesis" effect of the regulatory measures of the environmental protection tax law in the post-pandemic era
Source: PLoS One. 2024 May 31;19(5):e0304636. doi: 10.1371/journal.pone.0304636 (PMC11142481; doi:10.1371/journal.pone.0304636)
Supplement: S1 Data — (DOCX) [file pone.0304636.s001.docx]

Questionnaire

Hello, I would like to express my heartfelt thanks for taking time out of your busy schedule to participate in this survey. This survey mainly involves the contents of the environmental protection tax law of heavily polluting enterprises, which belongs to the academic research field. The purpose is to explore the environmental regulation effect of the environmental protection tax law on heavily polluting enterprises. The data obtained from this survey are strictly used for academic research, and the information of the individual or unit will never be disclosed. Please rest assured!

**[Filling Explanation]** This survey is designed to evaluate the regulatory effect of the environmental protection tax law, the green technology innovation ability of heavily polluting enterprises, and the competitive advantage of heavily polluting enterprises.The questionnaire was divided into seven grades according to the seven-point scale of Likert.Please choose the importance or conformity degree of the respondents under different items according to the experience, and type "√" on the corresponding grade scale.

## **Basic information**

1. Where you and your organization are located:

A. North China B. South China C. East China D. Southeast China E. Northeast China F. Northwest China G. Southwest China

1. Your industry:
2. Thermal Power B. Iron and Steel C. Cement D. Coal E. Chemical Industry F. Aluminum Smelting G. Metallurgy H. Petrochemical I. Brewing J. Building Materials K. Pharmaceutical L. Papermaking M. Fermentation N. Leathermaking O. Textile P. Mining Q. Other
3. Property of your company:
4. State-owned enterprises B. Private enterprises C. Foreign-funded enterprises
5. The number of employees the company has:
6. <=500 B. 501-1000 C. 1001～1500 D. 1501～2000 E. 2001～2500 F. 2501～3000 G. >=3001
7. Company operating life (Years)
8. <=5 B. 6～10 C. 11～15 D. 16～20 E. 21～25 F. 25～30 G. >=31
9. Company operating profit (Million Yuan)
10. <=5 B. 5-10 C. 10-15 D. 15-20 E. 20-25 F. 25-30 G. >=30
11. Your understanding of the impact of environmental protection tax laws on company’s business
12. Not familiar B. Certain understanding C. Understanding but not thorough D. Thorough understanding

## **Questionnaire on Environmental protection tax regulation effect**

### Environmental Protection Tax Act tax fairness questionnaire

| **Items or indicators** | **Disagree ←--------→ Agree**  **1 2 3 4 5 6 7** | | | | | | |
| --- | --- | --- | --- | --- | --- | --- | --- |
| The collection of environmental protection tax is fair to all enterprises in the industry. | □ | □ | □ | □ | □ | □ | □ |
| The collection of environmental protection tax by tax personnel is fair to all enterprises in the industry. | □ | □ | □ | □ | □ | □ | □ |
| Tax paying enterprises are fairness in understanding all kinds of tax information of tax collection institutions. | □ | □ | □ | □ | □ | □ | □ |
| The preferential reduction of environmental protection tax is fair to any industry in any industry or industry. | □ | □ | □ | □ | □ | □ | □ |

### **Environmental Protection Tax Act tax rationality** **questionnaire**

| **Items or indicators** | **Disagree ←--------→ Agree**  **1 2 3 4 5 6 7** | | | | | | |
| --- | --- | --- | --- | --- | --- | --- | --- |
| The setting level of the tax rate of the tax collection department in the region and the industry is reasonable. | □ | □ | □ | □ | □ | □ | □ |
| The environmental protection department is reasonable for measuring the discharge of waste water, waste gas and waste residue. | □ | □ | □ | □ | □ | □ | □ |
| Tax authorities for the accounting of the enterprise taxable amount is reasonable. | □ | □ | □ | □ | □ | □ | □ |
| The disclosure of tax collection information of the taxation department is reasonable in terms of its content, time and method. | □ | □ | □ | □ | □ | □ | □ |

## **Questionnaire on green technology innovation capability of heavy polluting enterprises**

### **Green process innovation capability of heavy polluting enterprises questionnaire**

| **Items or indicators** | **Disagree ←--------→ Agree**  **1 2 3 4 5 6 7** | | | | | | |
| --- | --- | --- | --- | --- | --- | --- | --- |
| Enterprise raw material procurement in the low-carbon environmental protection on the continuous innovation. | □ | □ | □ | □ | □ | □ | □ |
| Enterprise product production process in the low-carbon environmental protection on the continuous innovation. | □ | □ | □ | □ | □ | □ | □ |
| The use and transformation of production equipment in low carbon environmental protection innovation. | □ | □ | □ | □ | □ | □ | □ |
| The design, implementation and development of enterprise product sales channels are constantly innovating in low carbon environmental protection. | □ | □ | □ | □ | □ | □ | □ |

### **Green product innovation capability of heavy polluting enterprises questionnaire**

| **Items or indicators** | **Disagree ←--------→ Agree**  **1 2 3 4 5 6 7** | | | | | | |
| --- | --- | --- | --- | --- | --- | --- | --- |
| The performance of enterprise products is more conducive to environmental protection, energy conservation and emission reduction. | □ | □ | □ | □ | □ | □ | □ |
| Enterprise products are more conducive to environmental protection in the process of consumption or use. | □ | □ | □ | □ | □ | □ | □ |
| Enterprise products in the consumption or use of the process is more conducive to energy conservation and emission reduction. | □ | □ | □ | □ | □ | □ | □ |
| The emergence of enterprise products can guide or deepen the green concept of the public. | □ | □ | □ | □ | □ | □ | □ |

### **waste management innovation capability of heavy polluting enterprises questionnaire**

| **Items or indicators** | **Disagree ←--------→ Agree**  **1 2 3 4 5 6 7** | | | | | | |
| --- | --- | --- | --- | --- | --- | --- | --- |
| Water pollutant discharge treatment has a strong innovation ability. | □ | □ | □ | □ | □ | □ | □ |
| Air pollutant discharge treatment has a strong innovation ability. | □ | □ | □ | □ | □ | □ | □ |
| Solid pollutant discharge treatment has a strong innovation ability. | □ | □ | □ | □ | □ | □ | □ |
| The method or effect of noise control has a strong innovation ability. | □ | □ | □ | □ | □ | □ | □ |

## **Questionnaire on competitive advantage of heavy polluting enterprises**

| **Items or indicators** | **Disagree ←--------→ Agree**  **1 2 3 4 5 6 7** | | | | | | |
| --- | --- | --- | --- | --- | --- | --- | --- |
| The enterprise has a strong competitive advantage in the industry. | □ | □ | □ | □ | □ | □ | □ |
| The enterprise has a strong competitive advantage in the region. | □ | □ | □ | □ | □ | □ | □ |
| The enterprise has a strong competitive advantage in China. | □ | □ | □ | □ | □ | □ | □ |
| The enterprise has a strong competitive advantage in the world. | □ | □ | □ | □ | □ | □ | □ |

## **Data**

The symbols in the table represent the following elements:

TF: Tax fairness

TR: Tax rationality

PC: Green process innovation capability

PD: Green product innovation capability

WT: Waste treatment innovation capability

AD: Competitive advantage

|  | ${TF}_{1}$ | ${TF}_{2}$ | ${TF}_{3}$ | ${TF}_{4}$ | ${TR}_{1}$ | ${TR}_{2}$ | ${TR}_{3}$ | ${TR}_{4}$ | ${PC}_{1}$ | ${PC}_{2}$ | ${PC}_{3}$ | ${PC}_{4}$ | $\mathrm{PD}_{1}$ | $\mathrm{PD}_{2}$ | $\mathrm{PD}_{3}$ | $\mathrm{PD}_{4}$ | $\mathrm{WT}_{1}$ | $\mathrm{WT}_{2}$ | $\mathrm{WT}_{3}$ | $\mathrm{WT}_{4}$ | $\mathrm{AD}_{1}$ | $\mathrm{AD}_{2}$ | $\mathrm{AD}_{3}$ | $\mathrm{AD}_{4}$ |
| --- | --- | --- | --- | --- | --- | --- | --- | --- | --- | --- | --- | --- | --- | --- | --- | --- | --- | --- | --- | --- | --- | --- | --- | --- |
| s1 | 2 | 1 | 2 | 3 | 3 | 1 | 1 | 2 | 1 | 3 | 1 | 2 | 2 | 1 | 3 | 2 | 1 | 1 | 2 | 1 | 1 | 2 | 1 | 2 |
| 2 | 4 | 3 | 4 | 3 | 4 | 5 | 4 | 3 | 3 | 4 | 5 | 4 | 4 | 5 | 4 | 3 | 3 | 4 | 5 | 3 | 4 | 3 | 3 | 4 |
| 3 | 1 | 2 | 1 | 3 | 1 | 1 | 1 | 2 | 2 | 1 | 1 | 3 | 2 | 1 | 1 | 2 | 2 | 2 | 1 | 3 | 2 | 3 | 2 | 3 |
| 4 | 5 | 6 | 7 | 7 | 6 | 5 | 7 | 4 | 7 | 7 | 5 | 7 | 7 | 6 | 7 | 7 | 5 | 3 | 5 | 4 | 6 | 5 | 7 | 5 |
| 5 | 1 | 2 | 3 | 2 | 1 | 3 | 3 | 1 | 1 | 3 | 1 | 3 | 1 | 2 | 1 | 2 | 2 | 2 | 2 | 1 | 1 | 1 | 2 | 2 |
| 6 | 6 | 7 | 5 | 7 | 7 | 5 | 4 | 7 | 7 | 6 | 7 | 5 | 7 | 5 | 6 | 7 | 5 | 6 | 5 | 4 | 6 | 5 | 4 | 5 |
| 7 | 4 | 3 | 5 | 5 | 6 | 4 | 3 | 5 | 5 | 6 | 4 | 3 | 6 | 5 | 6 | 3 | 6 | 5 | 5 | 4 | 3 | 6 | 7 | 5 |
| 8 | 3 | 4 | 4 | 6 | 5 | 6 | 5 | 5 | 6 | 6 | 3 | 4 | 6 | 4 | 4 | 6 | 3 | 4 | 4 | 3 | 4 | 3 | 4 | 4 |
| 9 | 3 | 3 | 5 | 4 | 6 | 5 | 6 | 3 | 4 | 3 | 6 | 4 | 5 | 5 | 3 | 6 | 5 | 6 | 5 | 4 | 6 | 4 | 5 | 4 |
| 10 | 6 | 5 | 4 | 6 | 5 | 7 | 6 | 5 | 3 | 7 | 7 | 4 | 7 | 7 | 5 | 6 | 6 | 5 | 4 | 7 | 5 | 5 | 6 | 4 |
| 11 | 5 | 5 | 4 | 3 | 7 | 7 | 6 | 5 | 6 | 5 | 4 | 5 | 4 | 5 | 6 | 6 | 4 | 5 | 4 | 5 | 3 | 6 | 5 | 5 |
| 12 | 3 | 2 | 3 | 1 | 3 | 2 | 1 | 3 | 3 | 1 | 2 | 1 | 2 | 3 | 3 | 2 | 1 | 2 | 1 | 2 | 4 | 3 | 1 | 1 |
| 13 | 7 | 6 | 3 | 5 | 4 | 6 | 3 | 5 | 6 | 6 | 5 | 4 | 3 | 5 | 5 | 4 | 5 | 6 | 4 | 6 | 3 | 5 | 4 | 5 |
| 14 | 3 | 2 | 5 | 6 | 4 | 2 | 3 | 4 | 5 | 5 | 3 | 4 | 5 | 2 | 4 | 5 | 2 | 2 | 4 | 3 | 5 | 3 | 2 | 3 |
| 15 | 2 | 3 | 1 | 2 | 1 | 1 | 2 | 3 | 1 | 4 | 2 | 1 | 3 | 3 | 3 | 3 | 3 | 4 | 3 | 3 | 2 | 2 | 3 | 3 |
| 16 | 3 | 4 | 5 | 5 | 4 | 3 | 5 | 4 | 4 | 5 | 3 | 3 | 4 | 3 | 5 | 4 | 3 | 5 | 3 | 4 | 3 | 5 | 4 | 3 |
| 17 | 4 | 5 | 3 | 5 | 4 | 5 | 3 | 5 | 5 | 4 | 4 | 3 | 5 | 3 | 5 | 4 | 4 | 3 | 5 | 4 | 5 | 3 | 5 | 4 |
| 18 | 1 | 2 | 5 | 4 | 3 | 2 | 3 | 2 | 4 | 3 | 4 | 3 | 3 | 2 | 3 | 3 | 4 | 3 | 4 | 3 | 2 | 4 | 4 | 3 |
| 19 | 4 | 4 | 5 | 3 | 5 | 4 | 3 | 5 | 5 | 4 | 4 | 3 | 5 | 4 | 3 | 5 | 5 | 3 | 4 | 5 | 4 | 4 | 3 | 4 |
| 20 | 5 | 7 | 6 | 3 | 5 | 4 | 7 | 3 | 7 | 7 | 6 | 7 | 5 | 6 | 6 | 7 | 5 | 4 | 5 | 4 | 6 | 4 | 5 | 6 |
| 21 | 5 | 6 | 5 | 4 | 4 | 5 | 5 | 6 | 5 | 3 | 4 | 5 | 3 | 4 | 5 | 3 | 5 | 5 | 3 | 3 | 4 | 5 | 4 | 5 |
| 22 | 5 | 6 | 6 | 5 | 4 | 3 | 4 | 5 | 3 | 5 | 4 | 3 | 5 | 5 | 3 | 4 | 3 | 4 | 5 | 5 | 3 | 4 | 3 | 5 |
| 23 | 2 | 3 | 3 | 2 | 4 | 5 | 3 | 3 | 3 | 4 | 4 | 3 | 3 | 3 | 5 | 4 | 4 | 3 | 2 | 4 | 5 | 3 | 4 | 5 |
| 24 | 5 | 5 | 6 | 3 | 5 | 4 | 4 | 5 | 5 | 3 | 6 | 5 | 4 | 5 | 5 | 4 | 3 | 3 | 5 | 4 | 3 | 5 | 4 | 3 |
| 25 | 4 | 5 | 5 | 4 | 3 | 4 | 3 | 5 | 4 | 3 | 5 | 5 | 4 | 3 | 5 | 4 | 4 | 3 | 4 | 5 | 4 | 5 | 3 | 4 |
| 26 | 3 | 3 | 3 | 5 | 4 | 5 | 3 | 4 | 5 | 3 | 4 | 5 | 3 | 5 | 4 | 5 | 3 | 5 | 5 | 4 | 3 | 4 | 5 | 4 |
| 27 | 6 | 5 | 7 | 7 | 3 | 4 | 5 | 7 | 5 | 7 | 7 | 6 | 7 | 6 | 7 | 6 | 4 | 6 | 7 | 6 | 5 | 7 | 7 | 5 |
| 28 | 3 | 5 | 4 | 5 | 3 | 5 | 5 | 4 | 3 | 5 | 4 | 5 | 3 | 5 | 4 | 5 | 5 | 5 | 4 | 3 | 4 | 3 | 4 | 3 |
| 29 | 1 | 3 | 2 | 3 | 1 | 3 | 2 | 1 | 2 | 1 | 1 | 2 | 1 | 3 | 5 | 4 | 4 | 3 | 3 | 2 | 3 | 4 | 3 | 4 |
| 30 | 3 | 4 | 5 | 5 | 4 | 4 | 3 | 5 | 4 | 5 | 3 | 4 | 5 | 3 | 5 | 5 | 2 | 2 | 3 | 4 | 2 | 3 | 4 | 3 |
| 31 | 4 | 5 | 4 | 5 | 4 | 3 | 5 | 4 | 5 | 3 | 5 | 4 | 3 | 5 | 4 | 5 | 4 | 4 | 5 | 4 | 3 | 5 | 4 | 5 |
| 32 | 5 | 6 | 5 | 4 | 7 | 3 | 5 | 4 | 6 | 5 | 5 | 4 | 4 | 5 | 3 | 4 | 4 | 5 | 4 | 5 | 3 | 4 | 5 | 3 |
| 33 | 4 | 5 | 4 | 3 | 5 | 4 | 3 | 4 | 5 | 4 | 4 | 5 | 5 | 3 | 3 | 5 | 5 | 6 | 4 | 5 | 3 | 4 | 3 | 4 |
| 34 | 5 | 6 | 5 | 4 | 3 | 5 | 4 | 6 | 5 | 4 | 3 | 3 | 5 | 4 | 5 | 4 | 4 | 3 | 3 | 4 | 3 | 5 | 3 | 4 |
| 35 | 2 | 1 | 2 | 3 | 1 | 1 | 1 | 1 | 2 | 3 | 1 | 3 | 1 | 1 | 1 | 3 | 2 | 3 | 1 | 2 | 3 | 1 | 2 | 1 |
| 36 | 4 | 5 | 6 | 4 | 3 | 5 | 4 | 3 | 5 | 5 | 4 | 3 | 4 | 5 | 3 | 4 | 3 | 2 | 3 | 4 | 3 | 4 | 4 | 3 |
| 37 | 7 | 7 | 6 | 7 | 5 | 7 | 6 | 5 | 6 | 5 | 6 | 5 | 7 | 4 | 6 | 7 | 4 | 5 | 6 | 4 | 6 | 5 | 7 | 4 |
| 38 | 4 | 5 | 3 | 5 | 5 | 4 | 3 | 5 | 4 | 3 | 4 | 5 | 3 | 3 | 5 | 4 | 3 | 5 | 4 | 3 | 5 | 3 | 3 | 5 |
| 39 | 1 | 2 | 1 | 3 | 2 | 2 | 3 | 1 | 1 | 2 | 1 | 2 | 1 | 2 | 3 | 1 | 3 | 1 | 2 | 3 | 3 | 3 | 3 | 2 |
| 40 | 4 | 5 | 4 | 3 | 5 | 5 | 4 | 3 | 5 | 4 | 4 | 3 | 5 | 4 | 5 | 3 | 2 | 3 | 4 | 3 | 3 | 3 | 2 | 3 |
| 41 | 4 | 5 | 4 | 4 | 5 | 3 | 3 | 5 | 4 | 3 | 5 | 3 | 3 | 5 | 4 | 4 | 5 | 6 | 5 | 4 | 3 | 5 | 4 | 5 |
| 42 | 3 | 5 | 3 | 4 | 3 | 4 | 3 | 5 | 3 | 4 | 4 | 4 | 5 | 3 | 5 | 4 | 6 | 5 | 5 | 4 | 7 | 6 | 7 | 5 |
| 43 | 4 | 3 | 5 | 4 | 3 | 3 | 5 | 4 | 5 | 3 | 5 | 4 | 5 | 4 | 3 | 4 | 5 | 4 | 5 | 3 | 4 | 3 | 4 | 4 |
| 44 | 2 | 3 | 3 | 2 | 4 | 2 | 3 | 3 | 2 | 4 | 3 | 2 | 3 | 4 | 2 | 4 | 5 | 2 | 3 | 2 | 3 | 4 | 3 | 4 |
| 45 | 5 | 6 | 4 | 4 | 5 | 3 | 5 | 5 | 6 | 5 | 4 | 5 | 4 | 3 | 3 | 4 | 3 | 5 | 4 | 3 | 4 | 5 | 3 | 4 |
| 46 | 5 | 5 | 6 | 3 | 4 | 5 | 4 | 3 | 5 | 4 | 6 | 5 | 4 | 3 | 5 | 5 | 4 | 5 | 4 | 5 | 3 | 4 | 5 | 5 |
| 47 | 4 | 3 | 5 | 4 | 5 | 5 | 4 | 3 | 5 | 4 | 5 | 3 | 5 | 4 | 5 | 3 | 5 | 5 | 4 | 5 | 3 | 4 | 5 | 5 |
| 48 | 6 | 7 | 5 | 6 | 7 | 6 | 7 | 7 | 6 | 7 | 6 | 7 | 5 | 7 | 6 | 6 | 5 | 5 | 4 | 5 | 7 | 5 | 4 | 5 |
| 49 | 3 | 2 | 3 | 1 | 1 | 1 | 2 | 3 | 1 | 3 | 1 | 2 | 2 | 3 | 1 | 3 | 1 | 2 | 1 | 2 | 2 | 1 | 2 | 1 |
| 50 | 1 | 1 | 2 | 3 | 2 | 3 | 1 | 3 | 2 | 1 | 2 | 3 | 2 | 3 | 1 | 2 | 3 | 2 | 1 | 2 | 1 | 2 | 3 | 3 |
| 51 | 3 | 4 | 5 | 3 | 3 | 4 | 5 | 4 | 4 | 5 | 3 | 2 | 5 | 4 | 3 | 4 | 4 | 5 | 3 | 4 | 3 | 5 | 5 | 3 |
| 52 | 5 | 5 | 6 | 5 | 4 | 4 | 3 | 4 | 3 | 5 | 6 | 4 | 5 | 3 | 4 | 4 | 5 | 5 | 6 | 4 | 5 | 4 | 3 | 5 |
| 53 | 4 | 3 | 5 | 4 | 3 | 5 | 3 | 4 | 5 | 5 | 4 | 3 | 5 | 4 | 5 | 3 | 3 | 5 | 4 | 3 | 3 | 4 | 5 | 5 |
| 54 | 2 | 3 | 2 | 3 | 3 | 3 | 2 | 3 | 3 | 4 | 2 | 3 | 4 | 2 | 3 | 3 | 4 | 5 | 4 | 5 | 4 | 3 | 3 | 3 |
| 55 | 3 | 4 | 3 | 5 | 4 | 2 | 3 | 4 | 3 | 4 | 3 | 4 | 4 | 3 | 3 | 3 | 4 | 2 | 5 | 4 | 3 | 4 | 2 | 4 |
| 56 | 4 | 5 | 4 | 3 | 4 | 4 | 5 | 5 | 3 | 4 | 5 | 3 | 5 | 4 | 5 | 3 | 3 | 5 | 5 | 4 | 3 | 4 | 3 | 4 |
| 57 | 3 | 4 | 5 | 3 | 5 | 3 | 5 | 5 | 4 | 4 | 3 | 5 | 5 | 5 | 4 | 5 | 6 | 5 | 5 | 6 | 7 | 5 | 4 | 5 |
| 58 | 5 | 5 | 5 | 6 | 3 | 4 | 6 | 3 | 5 | 4 | 4 | 3 | 5 | 4 | 3 | 4 | 3 | 4 | 5 | 4 | 3 | 4 | 5 | 4 |
| 59 | 3 | 3 | 5 | 2 | 5 | 4 | 3 | 3 | 5 | 4 | 5 | 3 | 4 | 5 | 4 | 3 | 5 | 5 | 4 | 5 | 3 | 5 | 4 | 5 |
| 60 | 4 | 3 | 4 | 4 | 3 | 5 | 4 | 5 | 4 | 3 | 5 | 4 | 3 | 4 | 3 | 4 | 3 | 2 | 2 | 2 | 1 | 1 | 1 | 2 |
| 61 | 5 | 7 | 6 | 7 | 5 | 6 | 5 | 5 | 7 | 6 | 7 | 5 | 6 | 7 | 6 | 5 | 5 | 5 | 4 | 5 | 6 | 4 | 7 | 5 |
| 62 | 4 | 6 | 5 | 5 | 4 | 3 | 5 | 4 | 5 | 6 | 5 | 4 | 5 | 3 | 4 | 5 | 5 | 5 | 4 | 5 | 3 | 5 | 4 | 5 |
| 63 | 7 | 6 | 7 | 5 | 4 | 7 | 3 | 5 | 4 | 7 | 7 | 5 | 7 | 7 | 5 | 7 | 6 | 6 | 5 | 7 | 6 | 7 | 7 | 5 |
| 64 | 6 | 5 | 4 | 5 | 5 | 4 | 4 | 3 | 5 | 4 | 5 | 3 | 3 | 5 | 4 | 3 | 3 | 4 | 2 | 3 | 5 | 4 | 3 | 3 |
| 65 | 6 | 5 | 5 | 7 | 6 | 7 | 4 | 5 | 5 | 4 | 5 | 6 | 7 | 5 | 7 | 7 | 4 | 5 | 3 | 5 | 4 | 5 | 5 | 4 |
| 66 | 4 | 5 | 5 | 5 | 4 | 5 | 3 | 5 | 3 | 3 | 3 | 5 | 4 | 4 | 5 | 3 | 5 | 4 | 4 | 5 | 4 | 5 | 3 | 4 |
| 67 | 5 | 5 | 5 | 4 | 5 | 3 | 5 | 4 | 3 | 5 | 4 | 3 | 4 | 5 | 3 | 5 | 4 | 5 | 4 | 3 | 5 | 3 | 4 | 3 |
| 68 | 5 | 6 | 5 | 4 | 7 | 3 | 5 | 4 | 5 | 4 | 5 | 5 | 5 | 4 | 3 | 5 | 3 | 4 | 5 | 3 | 4 | 4 | 3 | 4 |
| 69 | 2 | 3 | 1 | 2 | 3 | 2 | 1 | 2 | 1 | 2 | 1 | 2 | 4 | 1 | 1 | 2 | 2 | 2 | 1 | 1 | 2 | 3 | 2 | 3 |
| 70 | 3 | 3 | 4 | 3 | 5 | 3 | 5 | 4 | 5 | 4 | 3 | 5 | 5 | 4 | 3 | 4 | 5 | 6 | 5 | 4 | 3 | 5 | 4 | 4 |
| 71 | 5 | 4 | 6 | 5 | 3 | 4 | 5 | 5 | 5 | 4 | 4 | 3 | 5 | 4 | 5 | 5 | 6 | 5 | 5 | 4 | 4 | 6 | 5 | 5 |
| 72 | 1 | 2 | 2 | 1 | 1 | 2 | 1 | 2 | 1 | 2 | 1 | 2 | 3 | 2 | 1 | 2 | 3 | 2 | 2 | 3 | 1 | 3 | 2 | 2 |
| 73 | 5 | 6 | 5 | 3 | 4 | 6 | 7 | 5 | 7 | 5 | 4 | 6 | 3 | 5 | 4 | 5 | 5 | 6 | 5 | 7 | 6 | 7 | 5 | 4 |
| 74 | 4 | 3 | 5 | 4 | 3 | 5 | 5 | 4 | 3 | 4 | 5 | 4 | 3 | 5 | 3 | 4 | 3 | 5 | 3 | 3 | 5 | 5 | 3 | 4 |
| 75 | 4 | 5 | 5 | 4 | 4 | 3 | 5 | 3 | 5 | 4 | 3 | 4 | 5 | 3 | 4 | 5 | 5 | 5 | 4 | 5 | 3 | 4 | 5 | 5 |
| 76 | 5 | 5 | 5 | 4 | 5 | 3 | 4 | 5 | 4 | 3 | 3 | 4 | 4 | 4 | 5 | 4 | 5 | 5 | 4 | 5 | 3 | 4 | 5 | 5 |
| 77 | 3 | 1 | 3 | 4 | 2 | 1 | 2 | 1 | 2 | 1 | 1 | 1 | 1 | 2 | 3 | 2 | 1 | 1 | 2 | 1 | 3 | 2 | 2 | 3 |
| 78 | 4 | 2 | 5 | 4 | 5 | 3 | 3 | 5 | 4 | 4 | 3 | 4 | 5 | 3 | 5 | 4 | 7 | 5 | 6 | 4 | 5 | 7 | 6 | 5 |
| 79 | 3 | 2 | 3 | 2 | 3 | 2 | 4 | 3 | 3 | 3 | 4 | 4 | 3 | 4 | 3 | 3 | 3 | 5 | 4 | 5 | 3 | 4 | 5 | 3 |
| 80 | 7 | 6 | 7 | 7 | 5 | 7 | 6 | 7 | 7 | 5 | 5 | 4 | 4 | 7 | 6 | 5 | 5 | 4 | 4 | 5 | 5 | 3 | 5 | 4 |
| 81 | 5 | 5 | 4 | 5 | 6 | 4 | 3 | 5 | 4 | 4 | 3 | 5 | 4 | 3 | 5 | 4 | 5 | 4 | 5 | 4 | 3 | 5 | 4 | 3 |
| 82 | 4 | 4 | 5 | 3 | 3 | 3 | 3 | 5 | 5 | 5 | 4 | 3 | 5 | 4 | 4 | 5 | 4 | 3 | 3 | 4 | 5 | 3 | 4 | 4 |
| 83 | 4 | 3 | 5 | 4 | 5 | 3 | 5 | 4 | 3 | 5 | 4 | 3 | 5 | 4 | 5 | 4 | 3 | 5 | 4 | 5 | 3 | 3 | 4 | 5 |
| 84 | 4 | 5 | 4 | 3 | 4 | 4 | 4 | 3 | 5 | 3 | 5 | 3 | 3 | 5 | 4 | 3 | 4 | 5 | 5 | 4 | 5 | 3 | 4 | 5 |
| 85 | 3 | 5 | 4 | 4 | 3 | 5 | 4 | 3 | 3 | 4 | 5 | 4 | 3 | 5 | 4 | 4 | 3 | 4 | 5 | 3 | 4 | 5 | 3 | 4 |
| 86 | 6 | 7 | 7 | 5 | 6 | 4 | 7 | 5 | 6 | 7 | 6 | 7 | 5 | 6 | 7 | 6 | 5 | 5 | 6 | 5 | 4 | 3 | 4 | 5 |
| 87 | 5 | 6 | 4 | 5 | 5 | 5 | 3 | 4 | 5 | 4 | 5 | 4 | 3 | 5 | 4 | 4 | 6 | 5 | 6 | 5 | 6 | 4 | 3 | 5 |
| 88 | 7 | 5 | 6 | 4 | 7 | 7 | 6 | 5 | 7 | 7 | 5 | 5 | 7 | 6 | 5 | 7 | 6 | 5 | 7 | 5 | 6 | 4 | 5 | 7 |
| 89 | 4 | 5 | 4 | 5 | 3 | 4 | 5 | 3 | 4 | 5 | 5 | 6 | 4 | 3 | 4 | 4 | 3 | 3 | 2 | 4 | 2 | 3 | 3 | 3 |
| 90 | 5 | 5 | 4 | 5 | 5 | 3 | 5 | 4 | 3 | 5 | 4 | 5 | 4 | 5 | 4 | 3 | 2 | 2 | 3 | 3 | 4 | 5 | 3 | 4 |
| 91 | 4 | 4 | 5 | 4 | 3 | 5 | 4 | 5 | 4 | 5 | 3 | 4 | 5 | 4 | 3 | 4 | 5 | 4 | 4 | 5 | 3 | 5 | 4 | 3 |
| 92 | 1 | 2 | 3 | 1 | 2 | 1 | 2 | 2 | 1 | 2 | 1 | 2 | 3 | 2 | 3 | 1 | 3 | 3 | 2 | 3 | 1 | 2 | 3 | 2 |
| 93 | 3 | 2 | 1 | 2 | 1 | 1 | 1 | 2 | 1 | 2 | 3 | 3 | 2 | 1 | 1 | 2 | 1 | 2 | 1 | 3 | 3 | 2 | 1 | 1 |
| 94 | 4 | 5 | 5 | 4 | 5 | 3 | 4 | 5 | 4 | 3 | 5 | 4 | 3 | 5 | 4 | 3 | 3 | 5 | 5 | 4 | 4 | 4 | 5 | 4 |
| 95 | 3 | 4 | 5 | 3 | 4 | 5 | 3 | 4 | 5 | 4 | 3 | 4 | 5 | 5 | 4 | 3 | 6 | 5 | 5 | 6 | 7 | 5 | 4 | 5 |
| 96 | 5 | 5 | 6 | 5 | 4 | 3 | 4 | 5 | 4 | 4 | 5 | 5 | 3 | 5 | 4 | 4 | 3 | 5 | 3 | 4 | 5 | 3 | 3 | 4 |
| 97 | 6 | 5 | 6 | 5 | 5 | 4 | 3 | 5 | 4 | 3 | 5 | 5 | 5 | 6 | 5 | 5 | 3 | 3 | 2 | 4 | 5 | 3 | 4 | 5 |
| 98 | 3 | 4 | 3 | 3 | 3 | 4 | 5 | 4 | 3 | 2 | 4 | 5 | 3 | 4 | 4 | 3 | 3 | 2 | 5 | 4 | 3 | 4 | 4 | 3 |
| 99 | 2 | 3 | 2 | 4 | 2 | 3 | 3 | 3 | 2 | 2 | 2 | 3 | 2 | 3 | 2 | 2 | 3 | 2 | 2 | 4 | 3 | 2 | 2 | 3 |
| 100 | 3 | 2 | 2 | 3 | 4 | 5 | 3 | 4 | 5 | 4 | 4 | 3 | 4 | 4 | 3 | 4 | 4 | 3 | 4 | 3 | 5 | 4 | 3 | 4 |
| 101 | 5 | 5 | 4 | 5 | 3 | 5 | 4 | 3 | 4 | 5 | 3 | 4 | 4 | 5 | 3 | 5 | 3 | 4 | 5 | 3 | 4 | 3 | 4 | 4 |
| 102 | 4 | 3 | 4 | 3 | 5 | 4 | 3 | 4 | 5 | 5 | 4 | 3 | 5 | 4 | 5 | 4 | 4 | 4 | 5 | 4 | 4 | 3 | 5 | 4 |
| 103 | 3 | 4 | 5 | 3 | 4 | 3 | 5 | 4 | 3 | 3 | 3 | 4 | 5 | 5 | 4 | 4 | 5 | 3 | 4 | 3 | 4 | 5 | 3 | 4 |
| 104 | 4 | 4 | 5 | 3 | 4 | 3 | 5 | 4 | 3 | 4 | 5 | 3 | 5 | 4 | 3 | 5 | 3 | 4 | 5 | 3 | 4 | 5 | 4 | 5 |
| 105 | 5 | 5 | 4 | 3 | 4 | 5 | 3 | 4 | 5 | 3 | 5 | 4 | 5 | 4 | 3 | 4 | 5 | 3 | 4 | 3 | 3 | 4 | 5 | 7 |
| 106 | 5 | 4 | 5 | 3 | 4 | 5 | 4 | 5 | 5 | 4 | 5 | 5 | 4 | 5 | 5 | 4 | 4 | 5 | 4 | 4 | 4 | 5 | 5 | 4 |
| 107 | 5 | 3 | 4 | 5 | 3 | 4 | 5 | 7 | 6 | 7 | 7 | 7 | 5 | 4 | 7 | 6 | 5 | 6 | 4 | 5 | 4 | 5 | 3 | 4 |
| 108 | 4 | 5 | 4 | 5 | 4 | 5 | 5 | 4 | 3 | 5 | 4 | 3 | 4 | 5 | 6 | 4 | 4 | 5 | 3 | 5 | 4 | 5 | 5 | 4 |
| 109 | 5 | 5 | 4 | 5 | 4 | 5 | 3 | 4 | 5 | 3 | 4 | 5 | 4 | 5 | 3 | 5 | 3 | 2 | 5 | 3 | 3 | 4 | 3 | 4 |
| 110 | 1 | 1 | 2 | 1 | 1 | 2 | 1 | 3 | 1 | 2 | 3 | 3 | 1 | 1 | 2 | 2 | 2 | 2 | 1 | 3 | 2 | 3 | 2 | 2 |
| 111 | 3 | 4 | 5 | 3 | 3 | 4 | 3 | 4 | 5 | 4 | 3 | 4 | 5 | 3 | 4 | 5 | 5 | 6 | 5 | 4 | 4 | 5 | 4 | 5 |
| 112 | 2 | 3 | 4 | 3 | 2 | 3 | 2 | 3 | 4 | 3 | 3 | 2 | 3 | 4 | 2 | 3 | 2 | 3 | 2 | 3 | 4 | 2 | 3 | 2 |
| 113 | 5 | 6 | 5 | 4 | 3 | 5 | 4 | 5 | 3 | 4 | 5 | 5 | 4 | 3 | 4 | 5 | 5 | 6 | 5 | 4 | 3 | 5 | 4 | 5 |
| 114 | 6 | 5 | 5 | 4 | 7 | 6 | 7 | 5 | 6 | 4 | 7 | 7 | 7 | 6 | 5 | 4 | 6 | 5 | 5 | 4 | 7 | 6 | 5 | 5 |
| 115 | 3 | 4 | 5 | 3 | 4 | 3 | 4 | 4 | 3 | 3 | 4 | 5 | 3 | 4 | 5 | 3 | 3 | 4 | 5 | 4 | 4 | 3 | 4 | 4 |
| 116 | 7 | 6 | 5 | 7 | 6 | 7 | 5 | 4 | 5 | 7 | 6 | 5 | 7 | 5 | 6 | 7 | 3 | 6 | 5 | 7 | 6 | 7 | 5 | 4 |
| 117 | 3 | 5 | 4 | 3 | 5 | 5 | 3 | 4 | 3 | 4 | 5 | 4 | 3 | 4 | 5 | 4 | 3 | 5 | 4 | 3 | 5 | 5 | 3 | 4 |
| 118 | 4 | 5 | 4 | 5 | 3 | 4 | 5 | 5 | 4 | 3 | 4 | 5 | 3 | 4 | 3 | 5 | 4 | 5 | 4 | 5 | 3 | 4 | 5 | 5 |
| 119 | 5 | 5 | 4 | 5 | 3 | 4 | 5 | 5 | 3 | 5 | 4 | 3 | 4 | 5 | 3 | 4 | 5 | 5 | 4 | 5 | 3 | 4 | 4 | 5 |
| 120 | 5 | 5 | 4 | 6 | 3 | 5 | 4 | 5 | 3 | 5 | 3 | 4 | 5 | 3 | 5 | 4 | 5 | 5 | 4 | 5 | 3 | 5 | 4 | 5 |
| 121 | 1 | 2 | 1 | 3 | 2 | 1 | 2 | 1 | 1 | 2 | 1 | 2 | 3 | 2 | 1 | 2 | 2 | 2 | 1 | 3 | 2 | 1 | 2 | 1 |
| 122 | 3 | 4 | 2 | 3 | 5 | 4 | 3 | 3 | 3 | 5 | 4 | 3 | 4 | 3 | 4 | 5 | 3 | 4 | 2 | 3 | 4 | 4 | 3 | 3 |
| 123 | 4 | 5 | 3 | 4 | 3 | 5 | 4 | 3 | 5 | 4 | 3 | 4 | 3 | 5 | 5 | 4 | 4 | 5 | 3 | 4 | 3 | 5 | 4 | 3 |
| 124 | 5 | 5 | 6 | 4 | 5 | 4 | 3 | 5 | 3 | 5 | 4 | 5 | 3 | 5 | 4 | 5 | 4 | 5 | 5 | 4 | 3 | 3 | 4 | 5 |
| 125 | 3 | 5 | 4 | 1 | 3 | 2 | 5 | 1 | 2 | 1 | 1 | 1 | 3 | 2 | 2 | 3 | 2 | 1 | 2 | 2 | 3 | 2 | 2 | 1 |
| 126 | 4 | 5 | 4 | 5 | 4 | 3 | 3 | 3 | 5 | 4 | 3 | 5 | 4 | 3 | 5 | 4 | 5 | 3 | 3 | 4 | 5 | 3 | 3 | 4 |
| 127 | 5 | 7 | 5 | 6 | 3 | 4 | 5 | 4 | 7 | 7 | 6 | 5 | 5 | 7 | 5 | 7 | 6 | 7 | 5 | 7 | 7 | 5 | 4 | 6 |
| 128 | 3 | 5 | 5 | 4 | 3 | 4 | 5 | 4 | 3 | 3 | 4 | 4 | 5 | 3 | 4 | 5 | 4 | 3 | 5 | 5 | 5 | 4 | 3 | 5 |
| 129 | 6 | 5 | 7 | 6 | 7 | 5 | 4 | 5 | 7 | 6 | 5 | 4 | 7 | 5 | 6 | 5 | 5 | 4 | 7 | 6 | 5 | 6 | 5 | 5 |
| 130 | 1 | 2 | 2 | 1 | 2 | 2 | 3 | 4 | 3 | 2 | 3 | 3 | 3 | 2 | 3 | 2 | 3 | 3 | 4 | 4 | 2 | 3 | 2 | 3 |
| 131 | 4 | 3 | 2 | 4 | 5 | 3 | 4 | 5 | 3 | 4 | 5 | 4 | 3 | 4 | 5 | 4 | 4 | 5 | 4 | 5 | 5 | 4 | 6 | 5 |
| 132 | 3 | 2 | 5 | 4 | 3 | 5 | 4 | 3 | 4 | 5 | 4 | 3 | 4 | 5 | 3 | 5 | 5 | 5 | 4 | 3 | 5 | 4 | 6 | 5 |
| 133 | 5 | 3 | 4 | 5 | 4 | 5 | 3 | 4 | 5 | 3 | 4 | 5 | 3 | 5 | 3 | 5 | 3 | 2 | 3 | 2 | 3 | 4 | 5 | 3 |
| 134 | 2 | 2 | 1 | 2 | 1 | 2 | 3 | 2 | 1 | 1 | 2 | 2 | 1 | 2 | 3 | 3 | 3 | 2 | 3 | 1 | 2 | 2 | 3 | 1 |
| 135 | 4 | 4 | 3 | 5 | 3 | 4 | 3 | 4 | 3 | 4 | 3 | 5 | 4 | 3 | 3 | 3 | 3 | 2 | 5 | 6 | 4 | 2 | 3 | 4 |
| 136 | 5 | 5 | 4 | 3 | 6 | 3 | 4 | 3 | 5 | 4 | 3 | 4 | 3 | 4 | 5 | 4 | 5 | 3 | 4 | 5 | 3 | 4 | 4 | 3 |
| 137 | 4 | 4 | 3 | 5 | 3 | 4 | 3 | 4 | 5 | 3 | 4 | 3 | 4 | 5 | 4 | 3 | 3 | 4 | 5 | 5 | 4 | 4 | 5 | 4 |
| 138 | 3 | 2 | 3 | 4 | 2 | 3 | 4 | 3 | 5 | 3 | 2 | 3 | 4 | 5 | 3 | 4 | 4 | 5 | 3 | 5 | 4 | 5 | 3 | 5 |
| 139 | 1 | 1 | 2 | 1 | 2 | 1 | 2 | 1 | 1 | 2 | 3 | 3 | 2 | 1 | 1 | 2 | 3 | 1 | 3 | 2 | 3 | 2 | 3 | 2 |
| 140 | 4 | 3 | 4 | 5 | 3 | 4 | 5 | 3 | 5 | 3 | 4 | 5 | 3 | 4 | 5 | 3 | 3 | 5 | 3 | 4 | 3 | 5 | 3 | 4 |
| 141 | 5 | 6 | 4 | 5 | 5 | 4 | 3 | 4 | 5 | 4 | 4 | 5 | 3 | 4 | 5 | 3 | 3 | 5 | 4 | 3 | 4 | 3 | 4 | 5 |
| 142 | 4 | 3 | 5 | 4 | 3 | 5 | 3 | 4 | 3 | 4 | 4 | 3 | 5 | 3 | 3 | 4 | 2 | 3 | 2 | 4 | 3 | 3 | 3 | 3 |
| 143 | 6 | 5 | 7 | 7 | 3 | 7 | 5 | 6 | 5 | 7 | 5 | 7 | 7 | 5 | 7 | 6 | 7 | 4 | 5 | 6 | 5 | 5 | 6 | 7 |
| 144 | 3 | 2 | 3 | 4 | 3 | 4 | 3 | 2 | 3 | 4 | 4 | 3 | 4 | 3 | 4 | 5 | 3 | 5 | 4 | 5 | 3 | 5 | 4 | 3 |
| 145 | 4 | 5 | 3 | 4 | 3 | 4 | 3 | 4 | 3 | 5 | 3 | 4 | 5 | 3 | 4 | 3 | 4 | 3 | 4 | 3 | 5 | 4 | 3 | 4 |
| 146 | 3 | 5 | 4 | 3 | 5 | 3 | 4 | 5 | 4 | 5 | 4 | 5 | 3 | 4 | 3 | 4 | 3 | 4 | 5 | 3 | 4 | 3 | 4 | 4 |
| 147 | 2 | 3 | 2 | 4 | 3 | 3 | 3 | 3 | 2 | 2 | 2 | 3 | 2 | 3 | 2 | 2 | 4 | 5 | 5 | 3 | 4 | 3 | 5 | 4 |
| 148 | 3 | 2 | 2 | 3 | 4 | 5 | 3 | 4 | 5 | 4 | 4 | 5 | 4 | 4 | 3 | 4 | 4 | 5 | 4 | 3 | 4 | 5 | 3 | 4 |
| 149 | 5 | 5 | 4 | 5 | 3 | 5 | 4 | 3 | 4 | 5 | 3 | 4 | 4 | 5 | 3 | 5 | 5 | 4 | 5 | 3 | 5 | 5 | 4 | 5 |
| 150 | 4 | 3 | 4 | 3 | 5 | 4 | 3 | 3 | 5 | 5 | 4 | 3 | 5 | 4 | 5 | 4 | 5 | 3 | 5 | 5 | 3 | 4 | 5 | 7 |
| 151 | 3 | 4 | 5 | 3 | 4 | 3 | 5 | 4 | 3 | 4 | 3 | 4 | 5 | 5 | 4 | 4 | 5 | 5 | 3 | 3 | 4 | 4 | 5 | 4 |
| 152 | 4 | 4 | 5 | 3 | 4 | 3 | 5 | 4 | 3 | 2 | 5 | 3 | 5 | 4 | 3 | 5 | 3 | 3 | 4 | 5 | 3 | 4 | 5 | 3 |
| 153 | 5 | 5 | 4 | 3 | 4 | 5 | 3 | 4 | 5 | 3 | 5 | 4 | 5 | 4 | 3 | 4 | 5 | 4 | 6 | 4 | 5 | 4 | 3 | 5 |
| 154 | 5 | 4 | 5 | 3 | 4 | 5 | 4 | 5 | 3 | 4 | 5 | 5 | 4 | 5 | 5 | 4 | 4 | 5 | 4 | 1 | 3 | 2 | 5 | 1 |
| 155 | 5 | 3 | 4 | 5 | 3 | 4 | 5 | 7 | 6 | 7 | 7 | 7 | 5 | 4 | 7 | 6 | 4 | 5 | 4 | 5 | 4 | 4 | 7 | 6 |
| 156 | 3 | 5 | 4 | 5 | 4 | 5 | 5 | 4 | 3 | 5 | 4 | 3 | 4 | 5 | 3 | 4 | 5 | 4 | 5 | 3 | 3 | 4 | 5 | 4 |
| 157 | 5 | 5 | 4 | 6 | 4 | 5 | 3 | 4 | 5 | 3 | 4 | 5 | 4 | 5 | 3 | 5 | 3 | 5 | 5 | 4 | 3 | 4 | 5 | 5 |
| 158 | 2 | 1 | 2 | 1 | 1 | 2 | 1 | 2 | 1 | 2 | 3 | 3 | 1 | 1 | 2 | 2 | 1 | 2 | 1 | 2 | 1 | 3 | 2 | 2 |
| 159 | 3 | 5 | 5 | 3 | 3 | 4 | 3 | 4 | 5 | 4 | 3 | 4 | 5 | 3 | 4 | 5 | 4 | 5 | 5 | 5 | 4 | 4 | 5 | 5 |
| 160 | 2 | 3 | 4 | 3 | 3 | 3 | 2 | 3 | 4 | 3 | 3 | 2 | 3 | 4 | 2 | 3 | 4 | 6 | 4 | 5 | 5 | 5 | 3 | 4 |
| 161 | 5 | 6 | 4 | 4 | 3 | 5 | 4 | 5 | 3 | 4 | 5 | 5 | 4 | 3 | 4 | 5 | 5 | 5 | 6 | 4 | 7 | 7 | 6 | 5 |
| 162 | 2 | 2 | 2 | 1 | 1 | 1 | 2 | 2 | 1 | 1 | 1 | 2 | 3 | 1 | 1 | 2 | 2 | 1 | 1 | 2 | 3 | 2 | 1 | 3 |
| 163 | 5 | 6 | 5 | 4 | 4 | 5 | 4 | 5 | 3 | 4 | 5 | 5 | 4 | 3 | 4 | 5 | 5 | 4 | 4 | 5 | 5 | 3 | 5 | 4 |
| 164 | 6 | 5 | 5 | 4 | 6 | 6 | 7 | 5 | 6 | 4 | 7 | 7 | 7 | 6 | 5 | 4 | 4 | 5 | 5 | 4 | 3 | 5 | 4 | 3 |
| 165 | 3 | 4 | 4 | 3 | 4 | 3 | 4 | 4 | 3 | 3 | 4 | 5 | 3 | 4 | 5 | 3 | 3 | 4 | 3 | 4 | 5 | 4 | 3 | 3 |
| 166 | 7 | 6 | 5 | 7 | 6 | 7 | 5 | 4 | 5 | 7 | 5 | 5 | 7 | 5 | 6 | 7 | 6 | 5 | 5 | 5 | 6 | 7 | 7 | 6 |
| 167 | 3 | 5 | 4 | 3 | 5 | 5 | 3 | 4 | 2 | 4 | 5 | 4 | 3 | 4 | 5 | 4 | 6 | 5 | 4 | 5 | 3 | 4 | 5 | 5 |
| 168 | 4 | 5 | 4 | 3 | 3 | 4 | 5 | 5 | 4 | 3 | 4 | 5 | 3 | 4 | 3 | 5 | 5 | 5 | 4 | 6 | 3 | 5 | 4 | 5 |
| 169 | 5 | 5 | 3 | 5 | 3 | 4 | 5 | 5 | 3 | 5 | 4 | 3 | 4 | 5 | 3 | 4 | 3 | 3 | 4 | 5 | 5 | 4 | 3 | 3 |
| 170 | 5 | 4 | 4 | 6 | 3 | 5 | 4 | 5 | 3 | 5 | 3 | 4 | 5 | 3 | 5 | 4 | 5 | 5 | 4 | 5 | 4 | 4 | 5 | 5 |
| 171 | 1 | 2 | 1 | 3 | 3 | 1 | 2 | 1 | 1 | 2 | 1 | 2 | 3 | 2 | 1 | 2 | 2 | 3 | 2 | 1 | 3 | 2 | 2 | 3 |
| 172 | 3 | 4 | 2 | 3 | 5 | 4 | 3 | 3 | 4 | 5 | 4 | 3 | 4 | 3 | 4 | 5 | 4 | 5 | 3 | 3 | 4 | 4 | 4 | 5 |
| 173 | 4 | 5 | 3 | 4 | 3 | 5 | 6 | 3 | 5 | 4 | 3 | 4 | 3 | 5 | 5 | 4 | 3 | 4 | 2 | 3 | 4 | 4 | 3 | 3 |
| 174 | 5 | 3 | 5 | 3 | 5 | 3 | 5 | 4 | 3 | 5 | 4 | 3 | 5 | 4 | 5 | 4 | 4 | 5 | 4 | 4 | 3 | 5 | 4 | 3 |
| 175 | 4 | 3 | 4 | 3 | 4 | 4 | 4 | 3 | 5 | 3 | 5 | 3 | 3 | 5 | 4 | 3 | 5 | 4 | 6 | 4 | 5 | 4 | 3 | 5 |
| 176 | 5 | 3 | 4 | 4 | 3 | 5 | 4 | 3 | 3 | 4 | 5 | 4 | 3 | 5 | 4 | 4 | 3 | 5 | 4 | 5 | 3 | 4 | 3 | 3 |
| 177 | 6 | 7 | 7 | 5 | 5 | 4 | 7 | 5 | 6 | 7 | 6 | 7 | 5 | 6 | 7 | 6 | 4 | 5 | 4 | 5 | 4 | 4 | 3 | 3 |
| 178 | 5 | 6 | 4 | 5 | 5 | 5 | 3 | 4 | 5 | 4 | 5 | 4 | 4 | 5 | 4 | 4 | 5 | 5 | 7 | 6 | 7 | 5 | 4 | 5 |
| 179 | 7 | 5 | 6 | 5 | 7 | 7 | 6 | 5 | 6 | 7 | 5 | 5 | 7 | 6 | 5 | 7 | 5 | 6 | 7 | 6 | 4 | 5 | 7 | 6 |
| 180 | 3 | 5 | 4 | 5 | 3 | 4 | 5 | 3 | 4 | 5 | 5 | 6 | 4 | 3 | 4 | 4 | 4 | 3 | 2 | 4 | 5 | 3 | 3 | 5 |
| 181 | 5 | 5 | 4 | 5 | 5 | 4 | 5 | 4 | 3 | 5 | 4 | 5 | 4 | 5 | 4 | 3 | 2 | 2 | 5 | 4 | 3 | 5 | 4 | 3 |
| 182 | 4 | 4 | 5 | 4 | 3 | 5 | 4 | 3 | 4 | 4 | 3 | 4 | 5 | 4 | 3 | 4 | 5 | 3 | 4 | 5 | 4 | 5 | 3 | 3 |
| 183 | 1 | 2 | 3 | 1 | 2 | 1 | 2 | 3 | 1 | 2 | 1 | 2 | 3 | 2 | 3 | 1 | 2 | 3 | 1 | 2 | 1 | 2 | 3 | 2 |
| 184 | 3 | 2 | 1 | 2 | 1 | 2 | 1 | 2 | 1 | 2 | 3 | 3 | 2 | 1 | 1 | 2 | 3 | 2 | 3 | 1 | 3 | 1 | 3 | 2 |
| 185 | 5 | 5 | 3 | 5 | 3 | 4 | 5 | 5 | 3 | 5 | 4 | 3 | 4 | 5 | 3 | 4 | 5 | 4 | 4 | 3 | 6 | 3 | 4 | 3 |
| 186 | 5 | 5 | 4 | 6 | 3 | 5 | 4 | 5 | 3 | 5 | 3 | 4 | 5 | 4 | 5 | 4 | 4 | 4 | 3 | 5 | 3 | 4 | 3 | 4 |
| 187 | 1 | 2 | 1 | 2 | 2 | 1 | 2 | 1 | 1 | 2 | 1 | 2 | 3 | 2 | 1 | 2 | 3 | 2 | 3 | 4 | 2 | 2 | 1 | 3 |
| 188 | 3 | 3 | 2 | 3 | 5 | 4 | 3 | 3 | 3 | 5 | 4 | 3 | 4 | 3 | 4 | 5 | 4 | 3 | 5 | 3 | 5 | 4 | 5 | 3 |
| 189 | 4 | 5 | 3 | 4 | 3 | 4 | 4 | 3 | 5 | 4 | 3 | 4 | 3 | 5 | 5 | 4 | 4 | 3 | 4 | 5 | 3 | 4 | 5 | 3 |
| 190 | 5 | 5 | 6 | 4 | 5 | 4 | 3 | 4 | 3 | 5 | 4 | 5 | 3 | 5 | 4 | 5 | 5 | 6 | 4 | 5 | 5 | 4 | 3 | 4 |
| 191 | 3 | 5 | 4 | 1 | 3 | 3 | 5 | 1 | 2 | 1 | 1 | 1 | 3 | 2 | 2 | 3 | 2 | 1 | 1 | 1 | 2 | 2 | 3 | 2 |
| 192 | 4 | 3 | 4 | 5 | 4 | 3 | 3 | 3 | 5 | 4 | 3 | 5 | 4 | 3 | 5 | 4 | 6 | 5 | 7 | 6 | 3 | 7 | 5 | 6 |
| 193 | 6 | 7 | 5 | 6 | 3 | 4 | 5 | 4 | 7 | 7 | 6 | 5 | 5 | 7 | 5 | 7 | 7 | 5 | 5 | 6 | 6 | 6 | 5 | 7 |
| 194 | 3 | 5 | 5 | 4 | 3 | 4 | 5 | 4 | 3 | 4 | 4 | 4 | 5 | 4 | 4 | 5 | 4 | 5 | 3 | 4 | 4 | 5 | 3 | 4 |
| 195 | 6 | 5 | 7 | 6 | 7 | 5 | 4 | 6 | 7 | 6 | 5 | 4 | 7 | 5 | 6 | 5 | 3 | 5 | 4 | 4 | 5 | 3 | 4 | 5 |
| 196 | 6 | 7 | 7 | 5 | 5 | 4 | 7 | 5 | 6 | 7 | 6 | 7 | 5 | 6 | 7 | 6 | 7 | 5 | 7 | 6 | 6 | 5 | 7 | 6 |
| 197 | 5 | 6 | 6 | 5 | 5 | 5 | 3 | 4 | 5 | 4 | 5 | 4 | 3 | 5 | 4 | 4 | 5 | 4 | 5 | 6 | 3 | 4 | 5 | 4 |
| 198 | 7 | 5 | 6 | 4 | 7 | 7 | 6 | 5 | 6 | 7 | 5 | 5 | 7 | 6 | 5 | 7 | 6 | 5 | 5 | 5 | 7 | 6 | 7 | 7 |
| 199 | 4 | 3 | 4 | 5 | 3 | 4 | 5 | 3 | 4 | 5 | 5 | 6 | 4 | 3 | 4 | 4 | 6 | 5 | 3 | 6 | 3 | 5 | 4 | 5 |
| 200 | 5 | 5 | 4 | 5 | 5 | 3 | 4 | 4 | 3 | 5 | 4 | 5 | 4 | 5 | 4 | 3 | 4 | 5 | 4 | 3 | 3 | 2 | 2 | 4 |
| 201 | 4 | 4 | 5 | 4 | 3 | 5 | 4 | 3 | 5 | 5 | 3 | 4 | 5 | 4 | 3 | 4 | 4 | 3 | 2 | 4 | 3 | 3 | 4 | 5 |
| 202 | 1 | 2 | 2 | 1 | 2 | 1 | 2 | 2 | 1 | 2 | 1 | 2 | 3 | 2 | 3 | 1 | 3 | 2 | 2 | 1 | 2 | 3 | 2 | 3 |
| 203 | 3 | 2 | 1 | 2 | 1 | 1 | 1 | 2 | 1 | 2 | 3 | 3 | 2 | 2 | 1 | 2 | 2 | 2 | 1 | 3 | 2 | 2 | 1 | 2 |
| 204 | 5 | 5 | 4 | 5 | 3 | 4 | 5 | 5 | 3 | 4 | 4 | 3 | 4 | 5 | 3 | 4 | 3 | 3 | 4 | 5 | 4 | 3 | 3 | 4 |
| 205 | 5 | 5 | 4 | 6 | 3 | 3 | 4 | 5 | 3 | 5 | 3 | 4 | 5 | 3 | 5 | 4 | 3 | 4 | 3 | 4 | 3 | 4 | 3 | 4 |
| 206 | 2 | 2 | 1 | 3 | 2 | 1 | 2 | 1 | 1 | 2 | 1 | 2 | 3 | 2 | 1 | 2 | 1 | 1 | 2 | 3 | 1 | 2 | 1 | 1 |
| 207 | 3 | 5 | 4 | 4 | 3 | 4 | 5 | 4 | 3 | 3 | 4 | 4 | 5 | 3 | 4 | 5 | 4 | 4 | 3 | 5 | 3 | 4 | 3 | 4 |
| 208 | 6 | 5 | 7 | 6 | 7 | 5 | 4 | 5 | 7 | 6 | 5 | 4 | 7 | 4 | 6 | 5 | 3 | 5 | 4 | 7 | 9 | 7 | 5 | 5 |
| 209 | 1 | 2 | 2 | 1 | 3 | 2 | 3 | 2 | 3 | 2 | 3 | 3 | 3 | 2 | 3 | 2 | 3 | 3 | 2 | 3 | 4 | 3 | 2 | 2 |
| 210 | 4 | 3 | 5 | 4 | 5 | 3 | 4 | 5 | 3 | 4 | 5 | 4 | 3 | 4 | 5 | 4 | 4 | 3 | 4 | 5 | 3 | 4 | 5 | 3 |
| 211 | 3 | 2 | 5 | 4 | 3 | 5 | 4 | 3 | 4 | 5 | 4 | 3 | 4 | 4 | 3 | 5 | 5 | 6 | 5 | 5 | 5 | 4 | 3 | 4 |
| 212 | 5 | 3 | 4 | 5 | 4 | 5 | 3 | 3 | 5 | 3 | 4 | 5 | 3 | 5 | 3 | 5 | 4 | 3 | 5 | 4 | 3 | 5 | 3 | 4 |
| 213 | 1 | 3 | 1 | 2 | 1 | 2 | 3 | 2 | 1 | 1 | 2 | 2 | 1 | 2 | 3 | 3 | 1 | 3 | 2 | 1 | 2 | 2 | 1 | 2 |
| 214 | 4 | 4 | 3 | 5 | 3 | 3 | 3 | 4 | 3 | 4 | 3 | 3 | 4 | 3 | 3 | 3 | 3 | 3 | 3 | 4 | 3 | 4 | 3 | 2 |
| 215 | 4 | 5 | 4 | 3 | 6 | 3 | 4 | 3 | 5 | 4 | 3 | 4 | 3 | 4 | 5 | 4 | 4 | 5 | 3 | 4 | 3 | 5 | 3 | 4 |
| 216 | 4 | 3 | 5 | 5 | 3 | 4 | 3 | 4 | 5 | 3 | 4 | 3 | 4 | 5 | 4 | 3 | 3 | 5 | 4 | 3 | 5 | 3 | 4 | 5 |
| 217 | 5 | 5 | 4 | 5 | 5 | 3 | 5 | 4 | 3 | 5 | 4 | 5 | 5 | 5 | 4 | 3 | 2 | 3 | 2 | 2 | 2 | 3 | 3 | 3 |
| 218 | 4 | 4 | 5 | 4 | 3 | 5 | 4 | 3 | 4 | 4 | 3 | 4 | 5 | 4 | 3 | 4 | 3 | 3 | 2 | 3 | 4 | 5 | 3 | 4 |
| 219 | 1 | 2 | 2 | 1 | 2 | 1 | 2 | 2 | 1 | 2 | 1 | 2 | 3 | 2 | 3 | 1 | 2 | 2 | 1 | 2 | 3 | 2 | 2 | 1 |
| 220 | 3 | 2 | 1 | 2 | 1 | 1 | 1 | 2 | 1 | 2 | 2 | 3 | 2 | 1 | 1 | 2 | 1 | 1 | 1 | 3 | 2 | 2 | 3 | 1 |
| 221 | 5 | 5 | 4 | 4 | 3 | 5 | 5 | 5 | 3 | 5 | 4 | 3 | 4 | 5 | 3 | 4 | 3 | 4 | 5 | 3 | 4 | 3 | 5 | 4 |
| 222 | 5 | 5 | 4 | 5 | 3 | 5 | 4 | 5 | 3 | 5 | 3 | 4 | 5 | 3 | 5 | 4 | 4 | 4 | 5 | 3 | 4 | 3 | 5 | 4 |
| 223 | 2 | 2 | 1 | 3 | 2 | 1 | 2 | 1 | 1 | 2 | 1 | 2 | 3 | 2 | 1 | 2 | 1 | 2 | 2 | 3 | 1 | 2 | 2 | 3 |
| 224 | 3 | 5 | 4 | 4 | 3 | 4 | 5 | 4 | 3 | 3 | 4 | 4 | 5 | 3 | 4 | 5 | 5 | 4 | 5 | 3 | 4 | 5 | 4 | 5 |
| 225 | 6 | 5 | 7 | 6 | 7 | 5 | 4 | 5 | 7 | 6 | 5 | 6 | 7 | 5 | 6 | 5 | 5 | 3 | 4 | 5 | 6 | 4 | 5 | 7 |
| 226 | 1 | 2 | 2 | 1 | 3 | 2 | 3 | 3 | 3 | 2 | 3 | 3 | 3 | 2 | 3 | 2 | 4 | 5 | 4 | 5 | 4 | 5 | 5 | 4 |
| 227 | 2 | 2 | 1 | 2 | 1 | 1 | 1 | 2 | 1 | 2 | 3 | 3 | 2 | 1 | 1 | 2 | 3 | 2 | 2 | 2 | 1 | 1 | 1 | 2 |
| 228 | 5 | 5 | 4 | 5 | 3 | 4 | 6 | 5 | 3 | 5 | 4 | 3 | 4 | 5 | 3 | 4 | 4 | 5 | 4 | 5 | 3 | 4 | 5 | 5 |
| 229 | 5 | 3 | 4 | 6 | 3 | 5 | 4 | 5 | 3 | 5 | 3 | 4 | 5 | 3 | 5 | 4 | 5 | 5 | 4 | 6 | 3 | 5 | 4 | 5 |
| 230 | 1 | 2 | 1 | 3 | 2 | 1 | 2 | 1 | 1 | 2 | 1 | 2 | 3 | 2 | 1 | 2 | 1 | 2 | 1 | 3 | 2 | 1 | 2 | 1 |
| 231 | 3 | 4 | 2 | 3 | 5 | 4 | 3 | 3 | 3 | 5 | 4 | 3 | 2 | 3 | 4 | 5 | 3 | 4 | 2 | 3 | 5 | 4 | 3 | 3 |
| 232 | 4 | 5 | 3 | 4 | 3 | 4 | 4 | 3 | 5 | 4 | 3 | 4 | 3 | 5 | 5 | 4 | 4 | 5 | 3 | 3 | 3 | 5 | 4 | 3 |
| 233 | 5 | 5 | 6 | 5 | 5 | 4 | 3 | 5 | 3 | 5 | 4 | 5 | 3 | 5 | 4 | 5 | 5 | 5 | 6 | 4 | 5 | 4 | 3 | 5 |
| 234 | 3 | 4 | 4 | 1 | 3 | 2 | 5 | 1 | 2 | 1 | 1 | 1 | 3 | 2 | 2 | 3 | 3 | 4 | 4 | 1 | 3 | 2 | 5 | 1 |
| 235 | 3 | 5 | 4 | 5 | 4 | 3 | 3 | 3 | 5 | 4 | 3 | 5 | 4 | 3 | 5 | 4 | 4 | 5 | 4 | 5 | 4 | 3 | 3 | 3 |
| 236 | 6 | 5 | 7 | 6 | 5 | 5 | 4 | 5 | 7 | 6 | 5 | 4 | 7 | 5 | 6 | 5 | 6 | 5 | 6 | 5 | 7 | 5 | 4 | 5 |
| 237 | 1 | 2 | 2 | 1 | 3 | 2 | 3 | 4 | 2 | 2 | 3 | 3 | 3 | 2 | 3 | 2 | 4 | 2 | 2 | 4 | 3 | 2 | 3 | 4 |
| 238 | 4 | 3 | 2 | 4 | 4 | 3 | 4 | 5 | 3 | 4 | 5 | 4 | 3 | 4 | 5 | 4 | 4 | 4 | 2 | 4 | 5 | 3 | 4 | 5 |
| 239 | 3 | 2 | 5 | 4 | 3 | 5 | 4 | 3 | 4 | 5 | 5 | 3 | 4 | 5 | 3 | 5 | 3 | 2 | 5 | 4 | 3 | 5 | 4 | 3 |
| 240 | 5 | 3 | 3 | 5 | 4 | 5 | 3 | 4 | 5 | 3 | 4 | 5 | 3 | 5 | 3 | 5 | 5 | 3 | 4 | 5 | 4 | 5 | 3 | 4 |
| 241 | 2 | 2 | 1 | 2 | 1 | 2 | 3 | 2 | 2 | 1 | 2 | 2 | 1 | 2 | 3 | 3 | 2 | 2 | 1 | 2 | 1 | 2 | 3 | 2 |
| 242 | 4 | 4 | 3 | 4 | 3 | 4 | 3 | 4 | 3 | 4 | 3 | 5 | 4 | 3 | 3 | 3 | 4 | 4 | 3 | 5 | 3 | 4 | 3 | 4 |
| 243 | 5 | 3 | 4 | 3 | 6 | 3 | 4 | 3 | 5 | 4 | 3 | 4 | 3 | 4 | 5 | 4 | 5 | 5 | 4 | 3 | 6 | 3 | 4 | 3 |
| 244 | 4 | 4 | 3 | 5 | 3 | 4 | 3 | 4 | 5 | 3 | 4 | 3 | 4 | 5 | 3 | 3 | 4 | 5 | 3 | 5 | 3 | 4 | 3 | 4 |
| 245 | 3 | 2 | 3 | 4 | 2 | 3 | 4 | 3 | 5 | 4 | 2 | 3 | 4 | 5 | 3 | 4 | 3 | 2 | 3 | 4 | 2 | 3 | 4 | 3 |
| 246 | 1 | 1 | 2 | 2 | 2 | 1 | 2 | 1 | 1 | 2 | 3 | 3 | 2 | 1 | 1 | 2 | 1 | 1 | 2 | 3 | 2 | 1 | 2 | 1 |
| 247 | 4 | 3 | 4 | 5 | 4 | 4 | 5 | 3 | 5 | 3 | 4 | 5 | 3 | 4 | 5 | 3 | 4 | 3 | 4 | 5 | 3 | 4 | 5 | 3 |
| 248 | 5 | 5 | 4 | 5 | 5 | 4 | 3 | 4 | 5 | 4 | 4 | 5 | 3 | 4 | 5 | 3 | 5 | 4 | 6 | 4 | 5 | 4 | 3 | 5 |
| 249 | 5 | 3 | 5 | 4 | 3 | 5 | 3 | 4 | 3 | 4 | 4 | 3 | 5 | 3 | 3 | 4 | 3 | 5 | 4 | 3 | 3 | 5 | 5 | 4 |
| 250 | 6 | 5 | 7 | 7 | 4 | 7 | 5 | 6 | 5 | 7 | 5 | 7 | 7 | 5 | 7 | 6 | 4 | 5 | 6 | 5 | 5 | 6 | 6 | 7 |
| 251 | 3 | 2 | 4 | 4 | 3 | 4 | 3 | 2 | 3 | 4 | 4 | 3 | 4 | 3 | 4 | 5 | 5 | 4 | 5 | 4 | 4 | 3 | 5 | 4 |
| 252 | 4 | 5 | 3 | 4 | 3 | 5 | 3 | 4 | 3 | 4 | 3 | 4 | 5 | 3 | 4 | 3 | 3 | 5 | 5 | 4 | 3 | 4 | 5 | 4 |
| 253 | 3 | 5 | 4 | 3 | 4 | 3 | 4 | 5 | 4 | 5 | 4 | 5 | 3 | 4 | 3 | 4 | 6 | 5 | 5 | 3 | 4 | 5 | 4 | 5 |
| 254 | 3 | 3 | 2 | 4 | 2 | 3 | 3 | 3 | 2 | 2 | 2 | 3 | 2 | 3 | 2 | 2 | 4 | 2 | 3 | 2 | 4 | 2 | 3 | 2 |
| 255 | 3 | 2 | 2 | 3 | 4 | 5 | 3 | 4 | 4 | 4 | 4 | 3 | 4 | 4 | 3 | 4 | 5 | 6 | 4 | 5 | 5 | 5 | 3 | 4 |
| 256 | 5 | 5 | 4 | 5 | 3 | 5 | 4 | 3 | 4 | 5 | 3 | 4 | 4 | 5 | 3 | 4 | 4 | 5 | 3 | 4 | 5 | 4 | 3 | 5 |
| 257 | 6 | 4 | 5 | 7 | 5 | 4 | 7 | 7 | 5 | 7 | 4 | 6 | 7 | 5 | 4 | 5 | 7 | 5 | 7 | 5 | 6 | 5 | 5 | 6 |
| 258 | 3 | 2 | 2 | 2 | 1 | 1 | 1 | 2 | 1 | 2 | 3 | 3 | 2 | 1 | 1 | 2 | 2 | 1 | 3 | 2 | 2 | 1 | 1 | 1 |
| 259 | 5 | 5 | 4 | 5 | 3 | 5 | 5 | 5 | 3 | 5 | 4 | 3 | 4 | 5 | 3 | 4 | 4 | 4 | 5 | 4 | 3 | 5 | 4 | 3 |
| 260 | 5 | 5 | 4 | 4 | 3 | 5 | 4 | 5 | 3 | 5 | 3 | 4 | 5 | 3 | 5 | 4 | 3 | 5 | 3 | 4 | 4 | 5 | 3 | 4 |
| 261 | 1 | 2 | 1 | 2 | 2 | 1 | 2 | 1 | 1 | 2 | 1 | 2 | 3 | 2 | 1 | 2 | 3 | 2 | 1 | 2 | 1 | 1 | 1 | 2 |
| 262 | 3 | 5 | 5 | 4 | 3 | 4 | 5 | 4 | 3 | 3 | 3 | 4 | 5 | 3 | 4 | 5 | 5 | 5 | 4 | 5 | 3 | 4 | 5 | 5 |
| 263 | 6 | 5 | 7 | 6 | 7 | 5 | 5 | 5 | 7 | 6 | 5 | 4 | 7 | 5 | 6 | 5 | 5 | 5 | 4 | 6 | 3 | 5 | 4 | 4 |
| 264 | 1 | 2 | 2 | 1 | 2 | 2 | 3 | 4 | 3 | 2 | 3 | 3 | 3 | 2 | 3 | 2 | 1 | 2 | 1 | 3 | 2 | 1 | 2 | 1 |
| 265 | 3 | 3 | 1 | 2 | 1 | 1 | 1 | 2 | 1 | 2 | 3 | 3 | 2 | 1 | 1 | 2 | 1 | 2 | 2 | 1 | 3 | 2 | 2 | 1 |
| 266 | 5 | 5 | 4 | 5 | 3 | 4 | 5 | 5 | 3 | 2 | 4 | 3 | 4 | 5 | 3 | 4 | 3 | 5 | 2 | 3 | 4 | 3 | 3 | 2 |
| 267 | 5 | 5 | 4 | 6 | 3 | 5 | 4 | 4 | 3 | 5 | 3 | 4 | 5 | 3 | 5 | 4 | 3 | 4 | 5 | 4 | 5 | 4 | 3 | 4 |
| 268 | 1 | 2 | 1 | 2 | 2 | 1 | 2 | 1 | 1 | 2 | 1 | 2 | 3 | 2 | 1 | 2 | 1 | 1 | 1 | 2 | 3 | 2 | 2 | 1 |
| 269 | 3 | 3 | 2 | 3 | 5 | 4 | 3 | 3 | 3 | 5 | 4 | 3 | 4 | 3 | 4 | 5 | 5 | 5 | 4 | 6 | 3 | 5 | 4 | 5 |
| 270 | 4 | 5 | 3 | 4 | 3 | 5 | 4 | 3 | 5 | 4 | 5 | 4 | 3 | 5 | 5 | 4 | 4 | 5 | 4 | 3 | 5 | 3 | 4 | 3 |
| 271 | 5 | 5 | 6 | 4 | 5 | 5 | 3 | 5 | 3 | 5 | 4 | 5 | 3 | 5 | 4 | 5 | 3 | 4 | 3 | 3 | 5 | 4 | 3 | 3 |
| 272 | 3 | 5 | 4 | 2 | 3 | 2 | 5 | 1 | 2 | 1 | 1 | 1 | 3 | 2 | 2 | 3 | 4 | 5 | 3 | 4 | 3 | 5 | 4 | 3 |
| 273 | 4 | 5 | 4 | 5 | 4 | 3 | 3 | 3 | 5 | 4 | 3 | 4 | 4 | 3 | 5 | 4 | 5 | 5 | 6 | 4 | 5 | 4 | 3 | 5 |
| 274 | 6 | 5 | 7 | 5 | 7 | 5 | 4 | 5 | 7 | 6 | 5 | 4 | 7 | 5 | 6 | 5 | 7 | 5 | 5 | 6 | 5 | 6 | 5 | 7 |
| 275 | 1 | 2 | 2 | 1 | 3 | 2 | 3 | 4 | 3 | 2 | 3 | 3 | 3 | 2 | 3 | 2 | 4 | 5 | 4 | 5 | 4 | 3 | 3 | 3 |
| 276 | 3 | 3 | 2 | 4 | 5 | 3 | 4 | 5 | 3 | 4 | 5 | 4 | 3 | 4 | 5 | 4 | 6 | 5 | 5 | 6 | 7 | 5 | 4 | 5 |
| 277 | 3 | 2 | 5 | 4 | 3 | 5 | 4 | 3 | 4 | 5 | 4 | 5 | 4 | 5 | 3 | 5 | 5 | 4 | 3 | 5 | 3 | 4 | 5 | 4 |
| 278 | 5 | 3 | 4 | 5 | 4 | 5 | 3 | 4 | 5 | 4 | 4 | 5 | 3 | 5 | 3 | 5 | 3 | 4 | 5 | 3 | 4 | 5 | 5 | 5 |
| 279 | 3 | 5 | 5 | 4 | 3 | 5 | 5 | 4 | 3 | 3 | 4 | 4 | 5 | 3 | 4 | 5 | 5 | 6 | 4 | 5 | 3 | 4 | 5 | 5 |
| 280 | 6 | 5 | 6 | 6 | 7 | 5 | 4 | 5 | 7 | 6 | 5 | 4 | 7 | 5 | 6 | 5 | 5 | 5 | 4 | 6 | 3 | 5 | 4 | 5 |
| 281 | 1 | 2 | 2 | 1 | 3 | 2 | 3 | 4 | 3 | 2 | 4 | 3 | 3 | 2 | 3 | 2 | 4 | 2 | 4 | 3 | 2 | 2 | 2 | 3 |
| 282 | 3 | 3 | 2 | 4 | 5 | 3 | 4 | 5 | 3 | 4 | 5 | 4 | 3 | 4 | 5 | 4 | 3 | 4 | 2 | 3 | 5 | 4 | 3 | 3 |
| 283 | 3 | 2 | 5 | 4 | 4 | 5 | 4 | 3 | 4 | 5 | 4 | 3 | 4 | 5 | 3 | 5 | 4 | 5 | 3 | 4 | 3 | 5 | 4 | 3 |
| 284 | 4 | 3 | 4 | 5 | 4 | 5 | 3 | 4 | 5 | 3 | 4 | 5 | 3 | 5 | 3 | 5 | 3 | 2 | 3 | 4 | 3 | 2 | 4 | 3 |
| 285 | 2 | 2 | 1 | 2 | 1 | 2 | 2 | 2 | 1 | 1 | 2 | 2 | 1 | 2 | 3 | 3 | 2 | 2 | 2 | 1 | 1 | 2 | 1 | 1 |
| 286 | 4 | 3 | 3 | 5 | 3 | 4 | 3 | 4 | 3 | 4 | 3 | 5 | 4 | 3 | 3 | 3 | 5 | 5 | 4 | 5 | 6 | 4 | 3 | 5 |
| 287 | 3 | 5 | 4 | 3 | 6 | 3 | 4 | 3 | 5 | 4 | 3 | 4 | 3 | 4 | 5 | 4 | 4 | 4 | 5 | 3 | 3 | 3 | 3 | 5 |
| 288 | 4 | 4 | 3 | 5 | 3 | 4 | 3 | 4 | 4 | 3 | 4 | 3 | 4 | 5 | 4 | 3 | 4 | 3 | 5 | 4 | 5 | 3 | 5 | 4 |
| 289 | 3 | 2 | 3 | 4 | 2 | 3 | 4 | 3 | 5 | 3 | 2 | 3 | 4 | 4 | 3 | 4 | 4 | 5 | 4 | 3 | 4 | 5 | 4 | 3 |
| 290 | 1 | 1 | 2 | 3 | 2 | 1 | 2 | 1 | 1 | 2 | 2 | 3 | 2 | 1 | 1 | 2 | 2 | 1 | 2 | 2 | 3 | 1 | 2 | 3 |
| 291 | 4 | 3 | 5 | 5 | 3 | 4 | 5 | 3 | 5 | 3 | 4 | 5 | 3 | 4 | 5 | 3 | 6 | 5 | 5 | 3 | 4 | 4 | 5 | 3 |
| 292 | 5 | 5 | 4 | 5 | 5 | 4 | 3 | 4 | 5 | 4 | 4 | 5 | 3 | 4 | 5 | 3 | 5 | 6 | 4 | 5 | 5 | 5 | 3 | 4 |
| 293 | 4 | 3 | 5 | 4 | 4 | 5 | 3 | 4 | 3 | 4 | 4 | 3 | 5 | 3 | 3 | 4 | 5 | 5 | 6 | 4 | 5 | 4 | 6 | 5 |
| 294 | 6 | 5 | 6 | 7 | 3 | 7 | 5 | 6 | 5 | 7 | 5 | 7 | 7 | 5 | 7 | 6 | 7 | 5 | 6 | 5 | 6 | 7 | 5 | 6 |
| 295 | 3 | 2 | 3 | 4 | 3 | 3 | 3 | 2 | 3 | 4 | 4 | 3 | 4 | 3 | 4 | 5 | 5 | 5 | 4 | 5 | 5 | 3 | 5 | 4 |
| 296 | 3 | 5 | 3 | 4 | 3 | 5 | 3 | 4 | 3 | 5 | 3 | 4 | 5 | 3 | 4 | 3 | 4 | 4 | 5 | 4 | 5 | 5 | 4 | 3 |
| 297 | 3 | 5 | 4 | 3 | 5 | 3 | 4 | 4 | 4 | 5 | 4 | 5 | 3 | 4 | 3 | 4 | 3 | 4 | 3 | 4 | 5 | 5 | 4 | 3 |
| 298 | 2 | 3 | 2 | 3 | 2 | 3 | 3 | 3 | 2 | 2 | 2 | 3 | 2 | 3 | 2 | 2 | 3 | 2 | 1 | 2 | 1 | 1 | 1 | 2 |
| 299 | 3 | 2 | 2 | 3 | 4 | 5 | 3 | 4 | 5 | 4 | 4 | 3 | 3 | 4 | 3 | 4 | 4 | 5 | 5 | 4 | 5 | 3 | 4 | 5 |
| 300 | 2 | 2 | 1 | 2 | 1 | 1 | 1 | 2 | 1 | 2 | 3 | 3 | 2 | 1 | 1 | 2 | 2 | 1 | 1 | 2 | 2 | 1 | 2 | 1 |
| 301 | 5 | 5 | 4 | 5 | 5 | 4 | 5 | 5 | 3 | 5 | 4 | 3 | 4 | 5 | 3 | 4 | 5 | 5 | 6 | 5 | 4 | 3 | 4 | 5 |
| 302 | 5 | 5 | 4 | 6 | 3 | 5 | 4 | 5 | 3 | 5 | 3 | 4 | 5 | 3 | 5 | 4 | 6 | 5 | 6 | 5 | 5 | 4 | 3 | 5 |
| 303 | 1 | 2 | 1 | 3 | 2 | 1 | 2 | 1 | 1 | 2 | 2 | 2 | 3 | 2 | 1 | 2 | 2 | 3 | 3 | 2 | 1 | 1 | 2 | 1 |
| 304 | 3 | 4 | 2 | 3 | 5 | 4 | 3 | 3 | 3 | 4 | 4 | 3 | 4 | 3 | 4 | 5 | 2 | 2 | 2 | 4 | 2 | 3 | 3 | 3 |
| 305 | 4 | 5 | 3 | 4 | 3 | 3 | 4 | 3 | 5 | 4 | 3 | 4 | 3 | 5 | 5 | 4 | 3 | 2 | 2 | 3 | 4 | 5 | 3 | 4 |
| 306 | 5 | 5 | 6 | 4 | 3 | 4 | 3 | 5 | 3 | 5 | 4 | 5 | 3 | 5 | 4 | 5 | 5 | 5 | 4 | 5 | 3 | 4 | 4 | 3 |
| 307 | 3 | 5 | 4 | 1 | 3 | 2 | 5 | 1 | 2 | 1 | 1 | 1 | 3 | 2 | 3 | 3 | 1 | 3 | 3 | 3 | 2 | 1 | 2 | 1 |
| 308 | 4 | 5 | 4 | 5 | 4 | 3 | 3 | 3 | 5 | 4 | 3 | 4 | 4 | 3 | 5 | 4 | 3 | 4 | 5 | 3 | 4 | 3 | 5 | 4 |
| 309 | 6 | 5 | 7 | 6 | 7 | 5 | 5 | 5 | 7 | 6 | 5 | 4 | 7 | 5 | 6 | 5 | 6 | 4 | 5 | 6 | 7 | 6 | 5 | 4 |
| 310 | 2 | 2 | 2 | 1 | 3 | 2 | 3 | 4 | 3 | 2 | 3 | 3 | 3 | 2 | 3 | 2 | 5 | 5 | 4 | 3 | 3 | 5 | 3 | 4 |
| 311 | 4 | 3 | 2 | 4 | 5 | 4 | 4 | 5 | 3 | 4 | 5 | 4 | 3 | 4 | 5 | 4 | 3 | 2 | 3 | 4 | 2 | 3 | 4 | 4 |
| 312 | 3 | 2 | 5 | 4 | 3 | 5 | 4 | 3 | 4 | 5 | 4 | 3 | 4 | 5 | 4 | 5 | 3 | 3 | 4 | 5 | 5 | 4 | 5 | 5 |
| 313 | 5 | 3 | 4 | 5 | 4 | 5 | 3 | 3 | 5 | 3 | 4 | 5 | 3 | 5 | 3 | 5 | 4 | 3 | 4 | 5 | 3 | 4 | 5 | 3 |
| 314 | 2 | 2 | 1 | 2 | 1 | 2 | 3 | 2 | 1 | 1 | 2 | 2 | 1 | 2 | 3 | 3 | 2 | 2 | 1 | 1 | 2 | 1 | 3 | 3 |
| 315 | 4 | 4 | 3 | 5 | 3 | 4 | 3 | 4 | 3 | 4 | 3 | 5 | 4 | 4 | 3 | 3 | 4 | 3 | 5 | 4 | 3 | 5 | 3 | 5 |
| 316 | 5 | 5 | 4 | 3 | 6 | 3 | 4 | 5 | 5 | 4 | 3 | 4 | 3 | 4 | 5 | 4 | 3 | 5 | 5 | 5 | 3 | 5 | 5 | 6 |
| 317 | 4 | 5 | 3 | 5 | 3 | 4 | 3 | 4 | 5 | 3 | 4 | 3 | 4 | 5 | 4 | 3 | 3 | 2 | 3 | 4 | 3 | 4 | 3 | 2 |
| 318 | 3 | 2 | 3 | 4 | 2 | 3 | 4 | 3 | 4 | 3 | 2 | 3 | 4 | 5 | 3 | 4 | 4 | 5 | 3 | 4 | 3 | 5 | 3 | 4 |
| 319 | 1 | 1 | 2 | 3 | 2 | 1 | 2 | 1 | 1 | 2 | 3 | 2 | 2 | 1 | 1 | 2 | 3 | 2 | 1 | 2 | 2 | 1 | 1 | 2 |
| 320 | 4 | 3 | 4 | 5 | 3 | 4 | 5 | 3 | 4 | 3 | 4 | 5 | 3 | 4 | 5 | 3 | 5 | 3 | 4 | 4 | 5 | 3 | 4 | 3 |
| 321 | 5 | 5 | 6 | 4 | 5 | 4 | 3 | 4 | 3 | 5 | 4 | 5 | 3 | 5 | 4 | 5 | 3 | 5 | 4 | 3 | 4 | 5 | 3 | 4 |
| 322 | 3 | 5 | 4 | 1 | 3 | 2 | 2 | 1 | 2 | 1 | 1 | 1 | 3 | 2 | 2 | 3 | 5 | 5 | 4 | 5 | 3 | 5 | 4 | 3 |
| 323 | 4 | 3 | 4 | 5 | 4 | 3 | 3 | 3 | 5 | 4 | 3 | 5 | 4 | 3 | 5 | 4 | 4 | 3 | 4 | 3 | 5 | 4 | 3 | 4 |
| 324 | 5 | 7 | 5 | 6 | 5 | 4 | 5 | 4 | 7 | 7 | 6 | 5 | 5 | 7 | 5 | 7 | 6 | 7 | 7 | 5 | 6 | 5 | 7 | 6 |
| 325 | 3 | 5 | 5 | 4 | 3 | 4 | 5 | 4 | 3 | 3 | 4 | 4 | 5 | 4 | 4 | 5 | 4 | 4 | 5 | 3 | 4 | 3 | 4 | 4 |
| 326 | 6 | 5 | 7 | 6 | 6 | 5 | 4 | 5 | 7 | 6 | 5 | 4 | 7 | 5 | 6 | 5 | 5 | 5 | 6 | 7 | 6 | 5 | 7 | 7 |
| 327 | 6 | 7 | 6 | 5 | 6 | 4 | 7 | 5 | 6 | 7 | 6 | 7 | 5 | 6 | 7 | 6 | 5 | 6 | 5 | 7 | 6 | 5 | 6 | 5 |
| 328 | 5 | 6 | 4 | 5 | 5 | 5 | 3 | 4 | 4 | 4 | 5 | 4 | 3 | 5 | 4 | 4 | 5 | 3 | 4 | 5 | 3 | 4 | 5 | 5 |
| 329 | 7 | 5 | 6 | 4 | 7 | 7 | 6 | 5 | 6 | 7 | 5 | 5 | 7 | 6 | 5 | 7 | 7 | 5 | 6 | 5 | 6 | 5 | 5 | 6 |
| 330 | 4 | 5 | 4 | 5 | 3 | 5 | 5 | 3 | 4 | 5 | 5 | 6 | 4 | 3 | 4 | 4 | 5 | 5 | 4 | 5 | 4 | 5 | 3 | 4 |
| 331 | 5 | 5 | 4 | 5 | 6 | 3 | 5 | 4 | 3 | 5 | 4 | 5 | 4 | 5 | 4 | 3 | 3 | 5 | 5 | 4 | 4 | 3 | 4 | 5 |
| 332 | 4 | 3 | 5 | 4 | 3 | 5 | 4 | 3 | 4 | 5 | 3 | 4 | 5 | 4 | 3 | 4 | 3 | 4 | 5 | 3 | 3 | 4 | 3 | 4 |
| 333 | 1 | 2 | 3 | 1 | 2 | 1 | 2 | 2 | 1 | 2 | 1 | 2 | 3 | 2 | 2 | 1 | 2 | 1 | 2 | 2 | 2 | 1 | 2 | 2 |
| 334 | 3 | 2 | 1 | 2 | 1 | 1 | 1 | 2 | 1 | 2 | 2 | 3 | 2 | 1 | 1 | 2 | 2 | 1 | 1 | 1 | 1 | 2 | 2 | 3 |
| 335 | 5 | 5 | 4 | 5 | 3 | 4 | 5 | 5 | 4 | 5 | 4 | 3 | 4 | 5 | 3 | 4 | 4 | 3 | 5 | 4 | 5 | 5 | 4 | 3 |
| 336 | 5 | 5 | 4 | 6 | 3 | 5 | 5 | 5 | 3 | 5 | 3 | 4 | 5 | 3 | 5 | 4 | 6 | 4 | 5 | 4 | 4 | 5 | 4 | 5 |
| 337 | 1 | 2 | 1 | 2 | 2 | 1 | 2 | 1 | 1 | 2 | 1 | 2 | 3 | 2 | 1 | 2 | 3 | 2 | 3 | 1 | 2 | 1 | 2 | 3 |
| 338 | 3 | 4 | 5 | 4 | 3 | 4 | 5 | 4 | 3 | 3 | 4 | 4 | 5 | 3 | 4 | 5 | 5 | 5 | 4 | 3 | 4 | 3 | 5 | 3 |
| 339 | 5 | 5 | 7 | 6 | 7 | 5 | 4 | 5 | 7 | 6 | 5 | 4 | 7 | 5 | 6 | 5 | 3 | 4 | 5 | 3 | 3 | 4 | 5 | 4 |
| 340 | 3 | 2 | 1 | 2 | 1 | 1 | 1 | 2 | 1 | 2 | 2 | 3 | 2 | 1 | 1 | 2 | 1 | 1 | 1 | 2 | 2 | 2 | 3 | 1 |
| 341 | 5 | 5 | 4 | 5 | 3 | 4 | 5 | 5 | 3 | 4 | 4 | 3 | 4 | 5 | 3 | 4 | 4 | 3 | 5 | 4 | 3 | 5 | 3 | 4 |
| 342 | 5 | 5 | 4 | 6 | 4 | 5 | 4 | 5 | 3 | 5 | 3 | 4 | 5 | 3 | 5 | 4 | 2 | 3 | 2 | 3 | 3 | 3 | 2 | 3 |
| 343 | 1 | 2 | 1 | 2 | 2 | 1 | 2 | 1 | 1 | 2 | 1 | 2 | 3 | 2 | 1 | 2 | 3 | 2 | 1 | 2 | 2 | 2 | 3 | 2 |
| 344 | 3 | 4 | 2 | 3 | 3 | 4 | 3 | 3 | 3 | 5 | 4 | 3 | 4 | 3 | 4 | 5 | 4 | 5 | 4 | 3 | 4 | 4 | 5 | 4 |
| 345 | 4 | 5 | 4 | 4 | 3 | 5 | 4 | 3 | 5 | 4 | 3 | 4 | 3 | 5 | 5 | 4 | 3 | 4 | 5 | 3 | 5 | 3 | 5 | 5 |
| 346 | 5 | 4 | 6 | 4 | 5 | 4 | 3 | 5 | 3 | 5 | 4 | 5 | 3 | 5 | 4 | 5 | 5 | 5 | 5 | 4 | 3 | 4 | 6 | 3 |
| 347 | 3 | 5 | 4 | 1 | 3 | 2 | 3 | 1 | 2 | 1 | 1 | 1 | 3 | 2 | 2 | 3 | 2 | 3 | 2 | 3 | 2 | 1 | 1 | 1 |
| 348 | 4 | 3 | 4 | 5 | 4 | 3 | 3 | 3 | 5 | 4 | 3 | 5 | 4 | 3 | 5 | 4 | 4 | 3 | 4 | 4 | 3 | 5 | 4 | 5 |
| 349 | 6 | 5 | 7 | 6 | 7 | 5 | 4 | 5 | 7 | 6 | 5 | 4 | 7 | 6 | 6 | 5 | 5 | 7 | 6 | 4 | 5 | 6 | 5 | 5 |
| 350 | 1 | 2 | 2 | 1 | 2 | 2 | 3 | 4 | 3 | 2 | 3 | 3 | 3 | 2 | 3 | 2 | 4 | 6 | 5 | 5 | 4 | 3 | 5 | 4 |
| 351 | 4 | 3 | 3 | 4 | 5 | 3 | 4 | 5 | 3 | 4 | 5 | 4 | 3 | 4 | 5 | 4 | 5 | 4 | 5 | 5 | 4 | 3 | 3 | 5 |
| 352 | 3 | 2 | 5 | 4 | 3 | 4 | 4 | 3 | 4 | 5 | 4 | 3 | 4 | 5 | 3 | 5 | 6 | 5 | 4 | 5 | 5 | 4 | 4 | 3 |
| 353 | 5 | 3 | 4 | 4 | 4 | 5 | 3 | 4 | 5 | 3 | 4 | 5 | 3 | 5 | 3 | 5 | 6 | 5 | 5 | 4 | 3 | 4 | 4 | 5 |
| 354 | 3 | 5 | 5 | 4 | 3 | 4 | 5 | 4 | 3 | 3 | 4 | 4 | 5 | 3 | 4 | 5 | 4 | 5 | 5 | 5 | 4 | 5 | 3 | 5 |
| 355 | 4 | 5 | 6 | 6 | 7 | 5 | 4 | 5 | 7 | 6 | 5 | 4 | 7 | 5 | 6 | 5 | 5 | 6 | 5 | 7 | 5 | 7 | 5 | 4 |
| 356 | 1 | 2 | 2 | 1 | 3 | 2 | 3 | 3 | 3 | 2 | 3 | 3 | 3 | 2 | 3 | 2 | 5 | 4 | 5 | 2 | 2 | 3 | 2 | 4 |
| 357 | 4 | 3 | 2 | 4 | 4 | 3 | 4 | 5 | 3 | 4 | 5 | 4 | 3 | 4 | 5 | 4 | 4 | 3 | 4 | 4 | 3 | 4 | 5 | 5 |
| 358 | 3 | 2 | 3 | 4 | 3 | 5 | 4 | 3 | 4 | 5 | 4 | 3 | 4 | 5 | 3 | 5 | 3 | 3 | 4 | 3 | 5 | 3 | 5 | 4 |
| 359 | 6 | 5 | 5 | 6 | 7 | 5 | 4 | 5 | 7 | 6 | 5 | 4 | 7 | 5 | 6 | 5 | 5 | 4 | 4 | 5 | 3 | 4 | 5 | 5 |
| 360 | 1 | 2 | 2 | 1 | 3 | 2 | 3 | 2 | 3 | 2 | 3 | 3 | 3 | 2 | 3 | 2 | 1 | 2 | 2 | 1 | 1 | 2 | 1 | 2 |
| 361 | 4 | 3 | 3 | 4 | 5 | 3 | 4 | 5 | 3 | 4 | 5 | 4 | 3 | 4 | 5 | 4 | 5 | 6 | 5 | 3 | 4 | 6 | 7 | 5 |
| 362 | 3 | 4 | 5 | 4 | 3 | 5 | 4 | 3 | 4 | 5 | 4 | 3 | 4 | 5 | 3 | 5 | 4 | 3 | 5 | 4 | 3 | 5 | 5 | 4 |
| 363 | 5 | 3 | 4 | 3 | 4 | 5 | 3 | 4 | 5 | 3 | 4 | 5 | 3 | 5 | 3 | 5 | 4 | 3 | 5 | 4 | 4 | 3 | 5 | 3 |
| 364 | 2 | 2 | 1 | 2 | 1 | 2 | 2 | 2 | 1 | 1 | 2 | 2 | 1 | 2 | 3 | 1 | 1 | 2 | 2 | 2 | 1 | 3 | 2 | 2 |
| 365 | 3 | 4 | 3 | 5 | 3 | 4 | 3 | 4 | 3 | 4 | 3 | 5 | 4 | 3 | 3 | 3 | 3 | 4 | 3 | 4 | 3 | 4 | 5 | 3 |
| 366 | 5 | 3 | 4 | 3 | 6 | 3 | 4 | 3 | 5 | 4 | 3 | 4 | 3 | 4 | 5 | 4 | 4 | 3 | 5 | 4 | 5 | 3 | 3 | 5 |
| 367 | 3 | 4 | 3 | 5 | 3 | 4 | 3 | 4 | 5 | 3 | 4 | 3 | 4 | 5 | 4 | 3 | 3 | 4 | 3 | 4 | 3 | 2 | 4 | 3 |
| 368 | 3 | 2 | 3 | 4 | 2 | 3 | 4 | 3 | 5 | 3 | 4 | 3 | 4 | 5 | 3 | 4 | 5 | 6 | 4 | 6 | 5 | 4 | 3 | 4 |
| 369 | 1 | 1 | 2 | 3 | 2 | 1 | 2 | 1 | 1 | 2 | 3 | 2 | 2 | 1 | 1 | 2 | 2 | 1 | 2 | 1 | 1 | 2 | 3 | 2 |
| 370 | 4 | 3 | 4 | 5 | 4 | 4 | 5 | 3 | 5 | 3 | 4 | 5 | 3 | 4 | 5 | 3 | 4 | 4 | 5 | 3 | 3 | 3 | 3 | 5 |
| 371 | 5 | 5 | 4 | 5 | 5 | 4 | 3 | 4 | 5 | 4 | 4 | 5 | 3 | 4 | 5 | 3 | 4 | 3 | 5 | 4 | 5 | 3 | 5 | 4 |
| 372 | 4 | 3 | 5 | 4 | 3 | 5 | 5 | 4 | 3 | 4 | 4 | 3 | 5 | 3 | 3 | 4 | 4 | 5 | 4 | 3 | 4 | 4 | 4 | 3 |
| 373 | 6 | 5 | 7 | 7 | 3 | 7 | 5 | 6 | 5 | 7 | 5 | 7 | 7 | 6 | 7 | 6 | 7 | 5 | 6 | 6 | 7 | 5 | 7 | 7 |
| 374 | 3 | 3 | 3 | 4 | 3 | 5 | 3 | 2 | 3 | 4 | 4 | 3 | 4 | 3 | 4 | 4 | 2 | 4 | 2 | 4 | 4 | 4 | 3 | 2 |
| 375 | 5 | 5 | 3 | 4 | 3 | 5 | 3 | 4 | 3 | 5 | 3 | 4 | 5 | 3 | 4 | 3 | 5 | 5 | 4 | 5 | 5 | 5 | 3 | 4 |
| 376 | 3 | 5 | 4 | 4 | 5 | 3 | 4 | 5 | 4 | 5 | 4 | 5 | 3 | 4 | 3 | 4 | 3 | 5 | 3 | 4 | 4 | 3 | 3 | 4 |
| 377 | 2 | 3 | 2 | 4 | 3 | 3 | 3 | 3 | 2 | 2 | 2 | 3 | 2 | 3 | 2 | 2 | 3 | 2 | 2 | 2 | 3 | 3 | 2 | 3 |
| 378 | 3 | 2 | 2 | 3 | 4 | 5 | 3 | 4 | 5 | 4 | 4 | 3 | 4 | 3 | 3 | 4 | 5 | 5 | 4 | 5 | 4 | 3 | 5 | 4 |
| 379 | 3 | 2 | 1 | 2 | 1 | 1 | 1 | 2 | 1 | 2 | 3 | 2 | 2 | 1 | 1 | 2 | 2 | 3 | 1 | 1 | 2 | 2 | 1 | 3 |
| 380 | 4 | 5 | 3 | 5 | 3 | 4 | 3 | 4 | 5 | 3 | 4 | 3 | 4 | 5 | 4 | 3 | 4 | 5 | 3 | 4 | 3 | 5 | 3 | 4 |
| 381 | 4 | 2 | 3 | 4 | 2 | 3 | 4 | 3 | 5 | 3 | 2 | 3 | 4 | 5 | 3 | 4 | 3 | 4 | 5 | 4 | 4 | 3 | 5 | 4 |
| 382 | 1 | 2 | 2 | 3 | 2 | 1 | 2 | 1 | 1 | 2 | 3 | 3 | 2 | 1 | 1 | 2 | 2 | 1 | 1 | 2 | 2 | 3 | 2 | 1 |
| 383 | 4 | 3 | 4 | 5 | 4 | 4 | 5 | 3 | 5 | 3 | 4 | 5 | 3 | 4 | 5 | 3 | 3 | 4 | 4 | 3 | 4 | 5 | 3 | 4 |
| 384 | 5 | 6 | 4 | 3 | 5 | 4 | 3 | 4 | 5 | 4 | 4 | 5 | 3 | 4 | 5 | 3 | 5 | 5 | 6 | 5 | 4 | 3 | 4 | 5 |
| 385 | 4 | 3 | 5 | 4 | 3 | 5 | 3 | 4 | 3 | 5 | 4 | 3 | 5 | 3 | 3 | 4 | 6 | 5 | 6 | 5 | 5 | 4 | 3 | 4 |
| 386 | 6 | 5 | 7 | 5 | 4 | 7 | 5 | 6 | 5 | 7 | 5 | 7 | 7 | 5 | 7 | 6 | 7 | 6 | 6 | 5 | 6 | 7 | 7 | 7 |
| 387 | 3 | 2 | 3 | 4 | 5 | 4 | 3 | 2 | 3 | 4 | 4 | 3 | 4 | 3 | 4 | 5 | 4 | 5 | 5 | 4 | 3 | 3 | 4 | 4 |
| 388 | 4 | 5 | 5 | 4 | 3 | 5 | 3 | 4 | 3 | 5 | 3 | 4 | 5 | 3 | 4 | 3 | 3 | 4 | 3 | 4 | 3 | 4 | 3 | 5 |
| 389 | 4 | 5 | 4 | 3 | 5 | 3 | 4 | 5 | 4 | 5 | 4 | 5 | 3 | 4 | 3 | 4 | 4 | 5 | 3 | 4 | 3 | 5 | 3 | 4 |
| 390 | 3 | 3 | 2 | 4 | 2 | 3 | 3 | 3 | 2 | 2 | 2 | 3 | 2 | 3 | 2 | 2 | 3 | 5 | 4 | 3 | 5 | 3 | 4 | 5 |
| 391 | 3 | 2 | 3 | 3 | 4 | 5 | 3 | 4 | 5 | 4 | 4 | 3 | 4 | 4 | 3 | 4 | 4 | 3 | 5 | 4 | 5 | 4 | 3 | 3 |
| 392 | 5 | 4 | 4 | 5 | 3 | 5 | 4 | 3 | 4 | 5 | 3 | 4 | 4 | 5 | 3 | 5 | 3 | 5 | 3 | 3 | 4 | 5 | 3 | 4 |
| 393 | 3 | 3 | 4 | 3 | 5 | 4 | 3 | 4 | 5 | 5 | 4 | 3 | 5 | 4 | 5 | 4 | 5 | 5 | 4 | 5 | 3 | 5 | 4 | 3 |
| 394 | 3 | 4 | 5 | 3 | 5 | 3 | 5 | 4 | 3 | 3 | 3 | 4 | 5 | 5 | 4 | 4 | 4 | 3 | 4 | 3 | 5 | 4 | 3 | 4 |
| 395 | 4 | 4 | 5 | 4 | 4 | 3 | 5 | 4 | 3 | 4 | 5 | 3 | 5 | 4 | 3 | 5 | 3 | 4 | 5 | 3 | 4 | 3 | 5 | 4 |
| 396 | 5 | 6 | 4 | 3 | 4 | 5 | 3 | 4 | 5 | 3 | 5 | 4 | 5 | 4 | 3 | 4 | 4 | 4 | 5 | 3 | 4 | 3 | 4 | 4 |
| 397 | 5 | 4 | 5 | 4 | 4 | 5 | 4 | 5 | 3 | 4 | 5 | 5 | 4 | 5 | 5 | 4 | 5 | 5 | 4 | 3 | 4 | 5 | 3 | 4 |
| 398 | 3 | 3 | 4 | 5 | 3 | 4 | 5 | 7 | 6 | 7 | 7 | 7 | 5 | 4 | 7 | 6 | 5 | 7 | 5 | 6 | 6 | 7 | 5 | 6 |
| 399 | 1 | 1 | 2 | 3 | 2 | 1 | 2 | 1 | 3 | 2 | 2 | 3 | 2 | 1 | 1 | 2 | 1 | 3 | 2 | 2 | 1 | 1 | 2 | 1 |
| 400 | 4 | 3 | 4 | 5 | 3 | 4 | 5 | 4 | 5 | 3 | 4 | 5 | 3 | 4 | 5 | 3 | 4 | 5 | 5 | 4 | 3 | 3 | 4 | 5 |
